# Phosphorylation‐Facilitated CKB Lactylation At K11 By GCN5 Enhances Creatine Kinase Activity and Mitigates Neuronal Damage After Cerebral Ischemia‐Reperfusion

**DOI:** 10.1002/advs.202516663

**Published:** 2026-07-17

**Authors:** Chao Duan, Ruolin Zhang, Shihui Ding, Ying Feng, Tao Shen, Ziwei Wang, Wendai Bao, Ke Shui, Jun Qin, Jun Chen, Min Zhang, Zijing Ren, Dongmei Zhao, Xingrong Guo, Zhibing Ai, Xin Yang, Peiyang Zhou, Zhiqiang Dong

**Affiliations:** ^1^ Hubei Provincial Clinical Research Center of Central Nervous System Repair and Functional Reconstruction Taihe Hospital Hubei University of Medicine Shiyan Hubei China; ^2^ College of Biomedicine and Health College of Life Science and Technology Huazhong Agricultural University Wuhan Hubei China; ^3^ Hubei Provincial Clinical Research Center for Umbilical Cord Blood Hematopoietic Stem Cells Taihe Hospital Hubei University of Medicine Shiyan Hubei China; ^4^ Department of Neurology Taihe Hospital Hubei University of Medicine Shiyan Hubei China; ^5^ Department of Neurology Xiangyang No.1 People's Hospital Hubei University of Medicine Xiangyang Hubei China; ^6^ Department of Electrical and Electronic Engineering School of Engineering Cardiff University Cardiff UK; ^7^ Hubei Jiangxia Laboratory Wuhan Hubei China

**Keywords:** CKB, ischemic stroke, lactylation, phosphorylation, proteomics

## Abstract

Ischemia‐reperfusion (I/R) injury in stroke causes severe neuronal damage through oxidative stress and metabolic dysfunction. Beyond its metabolic role, lactate can induce lysine lactylation, but its impact on neuroprotection remains unclear. Here, we employed integrative proteomic, lactylomic, and phosphoproteomic profiling in a murine I/R model and identified creatine kinase B‐type (CKB) as a dual target of K11 lactylation and S199 phosphorylation. These modifications were found to enhance CKB enzymatic activity, promote phosphocreatine metabolism, suppress reactive oxygen species, and support neuronal survival under ischemic conditions. Mechanistically, the acetyltransferase GCN5 catalyzed K11 lactylation, while Sirt5 functioned as a delactylase. Notably, S199 phosphorylation facilitated K11 lactylation by promoting GCN5 recruitment, suggesting a hierarchical interplay between the two modifications. In vivo studies confirmed that disruption of either K11 lactylation or S199 phosphorylation impaired CKB function, exacerbated neuronal injury, and delayed functional recovery after stroke. These findings reveal a coordinated post‐translational modification mechanism that enhances CKB activity and confers neuroprotection in cerebral I/R injury, offering a potential therapeutic target for stroke intervention.

## Graphical abstract

This study uncovers hierarchical coordination between K11 lactylation and S199 phosphorylation of CKB in cerebral ischemia‐reperfusion injury. Such dual modifications potentiate CKB enzymatic function, remodel energy metabolism, alleviate oxidative stress and neuronal damage, and represent a viable therapeutic target for stroke treatment.

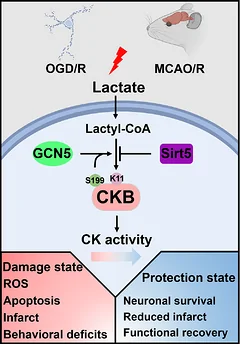

## 1 Introduction

Ischemic stroke, a leading cause of long‐term neurological disability and death worldwide, is characterized by abrupt cerebrovascular occlusion resulting in focal cerebral ischemia, metabolic failure, and neuronal loss [1, 2]. Accounting for approximately 87% of all stroke cases, ischemic stroke continues to rise in incidence, especially among aging populations [2]. The primary therapeutic approach, reperfusion therapy via thrombolysis or mechanical thrombectomy, aims to restore cerebral blood flow, but also introduces a double‐edged consequence: ischemia‐reperfusion (I/R) injury [3, 4, 5]. This pathological condition results from a sudden reintroduction of oxygen and nutrients following prolonged ischemia, initiating a cascade of oxidative stress, mitochondrial dysfunction, calcium overload, lipid peroxidation, and neuroinflammation [2, 6, 7, 8, 9, 10, 11]. Neurons, with high energy demands and limited reserves, are particularly vulnerable to such bioenergetic crises [12]. Thus, preserving neuronal metabolic integrity during I/R is an urgent unmet need in stroke therapy.

In recent years, lactate has gained attention as more than just a glycolytic byproduct. It functions as both an energy substrate and a metabolic signal in various physiological and pathological conditions, including tumorigenesis, myocardial remodeling, and neurodegeneration [13, 14, 15, 16, 17]. In the central nervous system, lactate is actively produced by astrocytes and shuttled to neurons, serving as an alternative fuel under energy stress [18, 19]. However, its role in stroke remains controversial. On one hand, exogenous lactate administration has been shown to ameliorate hypoxic‐ischemic injury [20, 21]; on the other, excessive intracellular lactate accumulation during ischemia may aggravate acidosis and neuronal damage [22, 23, 24]. Notably, lactate also contributes to epigenetic and post‐translational regulation via a newly discovered protein modification known as lysine lactylation [25]. This modification, in which a lactyl group is covalently attached to lysine residues, has been found to regulate histone dynamics, transcriptional activity, and cytosolic protein functions [26, 27]. In ischemic contexts, lactylation has been implicated in modulating neuronal apoptosis, glycolysis, inflammation, and mitochondrial crosstalk, highlighting its multifaceted roles in brain injury [28, 29, 30, 31, 32].

Despite these advances, the landscape of functionally relevant lactylation events during cerebral I/R remains largely unexplored. Especially lacking is our understanding of how lactylation affects core metabolic enzymes essential for neuronal survival under energy‐deprived conditions. One such enzyme is creatine kinase B‐type (CKB), a critical component of the creatine‐phosphocreatine (PCr) shuttle system that buffers intracellular ATP levels [33]. CKB is highly expressed in the brain and rapidly rephosphorylates ADP to ATP using PCr, thereby stabilizing energy supply during acute stress [34, 35]. Previous studies have demonstrated the neuroprotective potential of creatine supplementation in stroke models, but whether CKB activity is endogenously modulated by post‐translational modifications (PTMs) such as lactylation during I/R remains unknown [36, 37]. Given that lactate accumulates substantially during ischemia and that neurons rely on CKB for energy maintenance, we hypothesized that CKB may undergo functional lactylation as part of an adaptive neuroprotective response.

To test this hypothesis, we employed an integrative multi‐omics strategy, including quantitative lactylomics, phosphoproteomics, and proteomics in a murine middle cerebral artery occlusion/reperfusion (MCAO/R) model. Our analysis identified CKB as a prominent target of both lysine lactylation and serine phosphorylation during I/R. We further investigated the spatial‐temporal patterns of these modifications, their upstream regulators, and their effects on CKB enzymatic function and neuronal viability. In particular, we focused on the interplay between phosphorylation and lactylation, exploring whether hierarchical PTM crosstalk may regulate neuroprotective metabolism in the ischemic brain. This study provides mechanistic insight into lactate‐mediated protein regulation in neurons and reveals a novel metabolic signaling axis that may be leveraged for therapeutic intervention in stroke.

## 2 Results

### 2.1 Lactate Accumulation and Global Protein Lactylation are Elevated in Ischemic Models In Vivo and In Vitro

As schematized in Figure 1A, mice underwent transient MCAO followed by reperfusion, and ipsilateral cortices were harvested 24 h after reperfusion for lactate quantification and Pan‐Kla assessment by immunoblotting and immunofluorescence. In parallel, Neuro‐2a cells were subjected to OGD/R and analyzed using the same readouts. This two‐arm design tests whether I/R‐induced metabolic changes translate into proteome‐wide lactylation.

**FIGURE 1:**
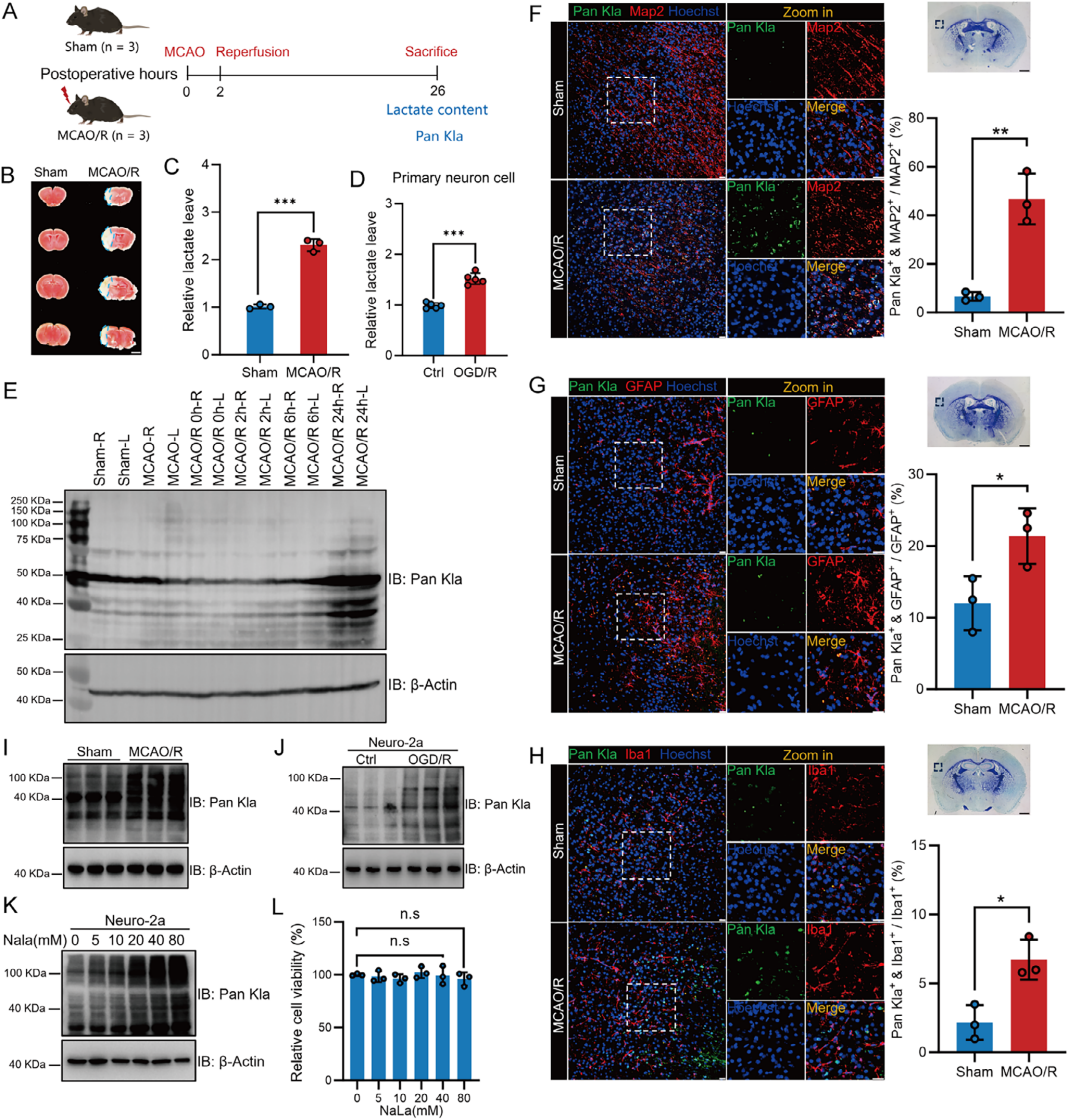
Lactate accumulation promotes protein lactylation in MCAO/R mice and OGD/R cellular models. (A) Experimental scheme for assessing lactate and global lysine lactylation: sham and MCAO/R mice were sampled 24 h after reperfusion, and the ipsilateral cortices were processed for lactate quantification and Pan‐Kla analysis. (B) Representative TTC‐stained coronal brain sections 24 h post‐MCAO/R, delineating infarct regions (dashed lines). *n* = 3 per group. Scale bar: 2 mm. (C) Quantitative analysis of cortical lactate levels in sham and MCAO/R mice. *n* = 3 per group. (D) Quantitative analysis of cortical lactate levels in Ctrl and OGD/R primary neuron cells. *n* = 5 per group. (E) Immunoblot analysis of global protein lactylation (Pan‐Kla) in ipsilateral (right) and contralateral (left) cortices from sham and MCAO/R mice at the indicated time points post MCAO/R. (F–H) Co‐immunofluorescence staining representative images (left panels) and quantification (right panels) of Pan‐Kla with MAP2 (neurons, F), GFAP (astrocytes, G), or Iba1 (microglia, H) in sham and MCAO/R cortices. *n* = 3 per group. Scale bar: 25 µm. (I) Western blot shows Pan‐Kla levels in ipsilateral cortices 24 h after MCAO/R. (J) Western blot shows Pan‐Kla levels in Neuro‐2a cells subjected to OGD/R or normoxic conditions. (K) Dose‐dependent induction of protein lactylation in Neuro‐2a cells treated with sodium lactate (0–80 mm, 24 h). (L) Cell viability assay of Neuro‐2a cells treated with NaLa (0–80 mm) for 24 h. *n* = 3 per group. Data normalized to untreated controls. Data are presented as mean ± SEM. Statistical significance was determined by one‐way ANOVA followed by Tukey's post hoc test. ^*^ *p* < 0.05, ^**^ *p* < 0.01, ^***^ *p* < 0.001.

The MCAO/R model was verified by 2,3,5‐triphenyl tetrazolium chloride (TTC) staining 24 h post‐reperfusion (Figure 1B). Colorimetric analysis revealed a significant increase in lactate levels in the ipsilateral cortex of MCAO/R mice and OGD/R primary neuron cells (Figure 1C,D), consistent with previous reports of lactate accumulation in ischemic brain tissue [24, 38]. Given that lactate serves as a substrate for lysine lactylation, we hypothesized that protein lactylation might participate in the ischemic response.

Western blot analysis revealed robust increases in global protein lactylation (Pan‐Kla) in the ipsilateral cortex following MCAO/R at multiple time points (Figure 1E). Immunofluorescence co‐staining further confirmed Pan‐Kla upregulation in neurons (MAP2^+^), astrocytes (GFAP^+^), and microglia (Iba1^+^) within ischemic brain regions (Figure 1F–H). Consistently, both MCAO/R mice and OGD/R cellular models exhibited markedly elevated protein lactylation levels, as demonstrated by immunoblotting (Figure 1I,J). Furthermore, exogenous sodium lactate (NaLa) treatment significantly increased lactylation levels without affecting cell viability (Figure 1K,L). Together, these findings indicate that I/R stress triggers lactate accumulation and protein lactylation, suggesting a potential regulatory role for lactylation in cerebral I/R pathophysiology.

### 2.2 Global Proteome, Lactylome, and Phosphoproteome Profiling Reveal Post‐Translational Reprogramming in Ischemic Cortex

To capture proteome‐wide remodeling, we profiled cortex from sham and MCAO/R mice (*n* = 3 per group) using label‐free proteomics together with enrichment‐based lactylome and phosphoproteome (Figure 2A). Principal component analysis (PCA) confirmed strong reproducibility among biological replicates (Figure S1A), and peptide features conformed to expected quality metrics (Figure S1B). A total of 87 differentially expressed proteins (DEPs) were identified by comparative proteomic analysis (59 upregulated and 28 downregulated) in MCAO/R mice compared to sham (Figure S1C,D). Volcano plot visualization highlights the up‐ and downregulated proteins (Figure S1E). Protein‐protein interaction (PPI) network analysis revealed proteins such as Alb, Fga, Apoa1, and Pig (Figure S1F), all previously linked to I/R injury [39, 40, 41]. Functional annotation based on COG/KOG indicated significant enrichment in biological processes, including post‐translational modification, protein turnover, defense, and signal transduction (Figure S1G), underscoring the multifaceted proteomic alterations elicited by I/R injury.

**FIGURE 2:**
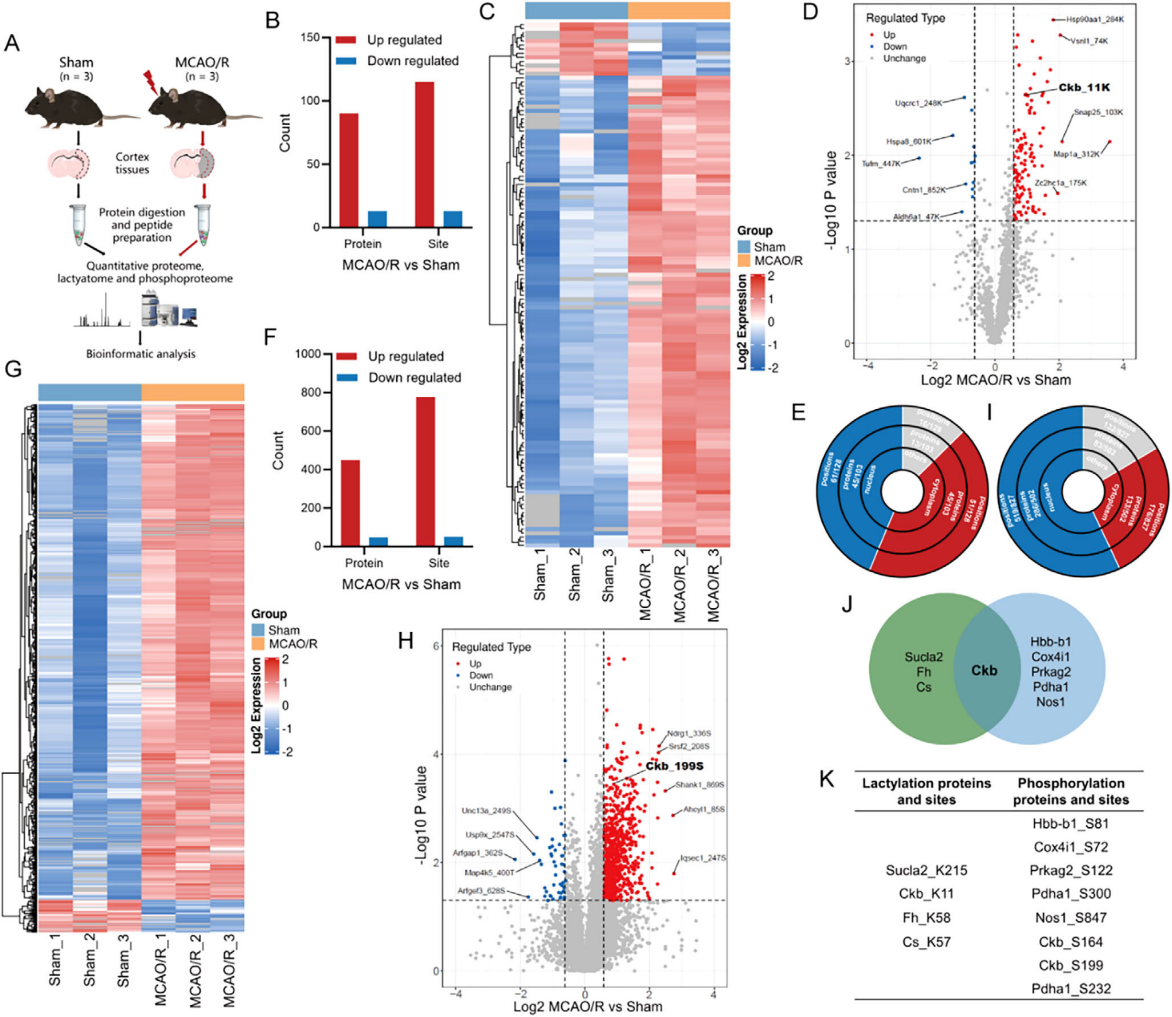
Quantitative profiling of protein lactylation and phosphorylation in MCAO/R mice cortex. (A) Schematic workflow of quantitative proteomic and lactylomic analysis in the mouse cortex following sham or MCAO/R surgery. (B) Number of significantly dysregulated lactylated peptides in MCAO/R vs. sham cortices (fold change > 1.5 or < 0.67, *p* < 0.05, *t*‐test). (C) Heatmaps of differentially modified peptides identified by lactylome. (D) Volcano plots of lactylated peptides. Red and blue dots indicate up‐ and downregulated modifications. (E) Subcellular localization of dysregulated lactylated proteins. (F) Number of significantly dysregulated phosphorylated peptides in MCAO/R vs. sham cortices (fold change > 1.5 or < 0.67, *p* < 0.05, *t*‐test). (G) Heatmaps of differentially modified peptides identified by phosphoproteome. (H) Volcano plots of phosphorylated peptides. Red and blue dots indicate up‐ and downregulated modifications. (I) Subcellular localization of dysregulated phosphorylated proteins. (J, K) Functional enrichment of lactylome and phosphoproteome differentially modified proteins (J) and sites (K) in the energy metabolism pathways (COG/KOG classification).

To further elucidate the regulatory landscape of protein modifications under I/R conditions, we conducted parallel lactylome and phosphoproteome analysis. PCA and peptide distribution profiling confirmed the high reproducibility and reliability of the lactylome and phosphoproteome data among biological replicates (Figures S2A,B and S3A,B). This revealed a total of 128 differentially lactylated lysine sites, including 115 upregulated and 13 downregulated sites in the MCAO/R cortices (Figure 2B). Heatmap and volcano plots displayed lactylation sites of proteins identified by lactylome (Figure 2C,D). These lactylated proteins were predominantly localized within the nucleus (45 out of 103) and cytoplasm (45 out of 103) (Figure 2E). Notably, proteins such as Snap25, Aldoc, Mapt, and Hnrnpu emerged as central nodes in the lactylome‐associated PPI network (Figure S2C). Sequence motif analysis of regions flanking the modified lysines indicated a preference for alanine, aspartate, glycine, and lysine residues (Supplemental Figure S2D), suggesting potential substrate specificity for lactylation under I/R stress. Upon these findings, phosphoproteomic profiling was performed to explore additional layers of post‐translational modifications under I/R conditions. Compared with sham groups, a total of 827 phosphorylation sites exhibited differential regulation in the cortex of MCAO/R mice, including 776 upregulated sites from 449 proteins and 51 downregulated sites from 47 proteins (Figure 2F). Heatmap and volcano plots illustrated the top 5 dysregulated phosphorylation sites (Figure 2G,H). Similar to lactylated proteins, phosphorylated targets were mainly distributed in nuclear (286 out of 502) and cytoplasmic (133 out of 502) compartments (Figure 2I). Amino acid frequency analysis surrounding the phosphorylated sites showed that serine, glutamate, proline, and aspartate were the most frequently occurring residues flanking the modification sites, while lysine and arginine were predominantly enriched upstream (Figure S3C). PPI network analysis identified Dlg4, Syngap1, Grin2b, and Gsk3b as significantly enriched nodes (Figure S3D).

To further characterize the interplay between the lactylome and phosphoproteome in MCAO/R cortices, we performed COG/KOG functional annotation on the sets of dysregulated lactylated and phosphorylated proteins, which highlighted overlapping enrichment in energy metabolism and cellular stress responses (Figure S4A,B). Notably, Venn analysis revealed CKB as a dually‐modified enzyme carrying both K11 lactylation and S199 phosphorylation (Figure 2J,K), nominating CKB as a convergent node. Given the critical role of CKB in maintaining energy balance and mediating ATP regeneration, we hypothesized that these two modifications act in concert to regulate metabolic homeostasis during I/R injury.

### 2.3 CKB K11 Lactylation Enhances Creatine Kinase Activity and Confers Neuroprotection Against OGD/R‐Induced Injury

Given the well‐established role of CKB in ATP regeneration via the phosphocreatine system [33, 34], we next investigated whether CKB function is modulated through lysine lactylation under ischemic conditions. Quantitative lactylome profiling revealed a marked increase in lactylation at lysine 11 (K11) of CKB in MCAO/R mouse cortices (Figure 3A). Q‐PCR data showed no significant difference in CKB mRNA between Ctrl and OGDR‐treated Neuro‐2a cells (Figure S2E). To investigate the functional significance of this modification, we generated and sequence‐verified Flag‐tagged wild‐type (Flag‐CKB) and K11R mutant (Flag‐CKB *^K11R^*) plasmids, in which the lysine residue was substituted with arginine to prevent lactylation (Figure 3B). These plasmids were transfected into Neuro‐2a cells, which were then subjected to oxygen‐glucose deprivation and reoxygenation (OGD/R) to mimic I/R conditions in vitro. Immunoprecipitation (IP) followed by Western blotting confirmed comparable expression of Flag‐CKB and Flag‐CKB *^K11R^* proteins in both control and OGD/R‐treated cells. Pan‐Kla was detected on Flag immunoprecipitates from cells expressing Flag‐CKB or Flag‐CKB *^K11R^*, revealing a marked reduction of Flag‐CKB *^K11R^* lactylation despite comparable expression (Figure 3C), validating CKB K11 as a bona fide lactylation site. Viability assessment of Neuro‐2a cells indicated that Flag‐CKB *^K11R^* failed to confer protection against OGD/R‐induced cytotoxicity, in contrast to CKB (Figure 3D). To determine the enzymatic consequences of K11 lactylation, we measured creatine kinase (CK) activity in transfected Neuro‐2a cells post‐OGD/R. Overexpression of Flag‐CKB significantly elevated CK activity compared to vector control, whereas the Flag‐CKB *^K11R^* abolished this enhancement (Figure 3E). Biochemical assays further demonstrated that Flag‐CKB‐expressing cells exhibited reduced intracellular creatine levels after OGD/R, indicative of accelerated phosphocreatine synthesis. Conversely, Flag‐CKB *^K11R^* cells displayed attenuated creatine consumption, consistent with reduced enzymatic activity (Figure 3F). Accordingly, intracellular reactive oxygen species (ROS) were detected using the DCF‐DA fluorescent probe. Flag‐CKB overexpression significantly suppressed ROS accumulation following OGD/R treatment, while the Flag‐CKB *^K11R^* mutant failed to provide such antioxidant protection and was associated with increased ROS levels (Figure 3G,H).

**FIGURE 3:**
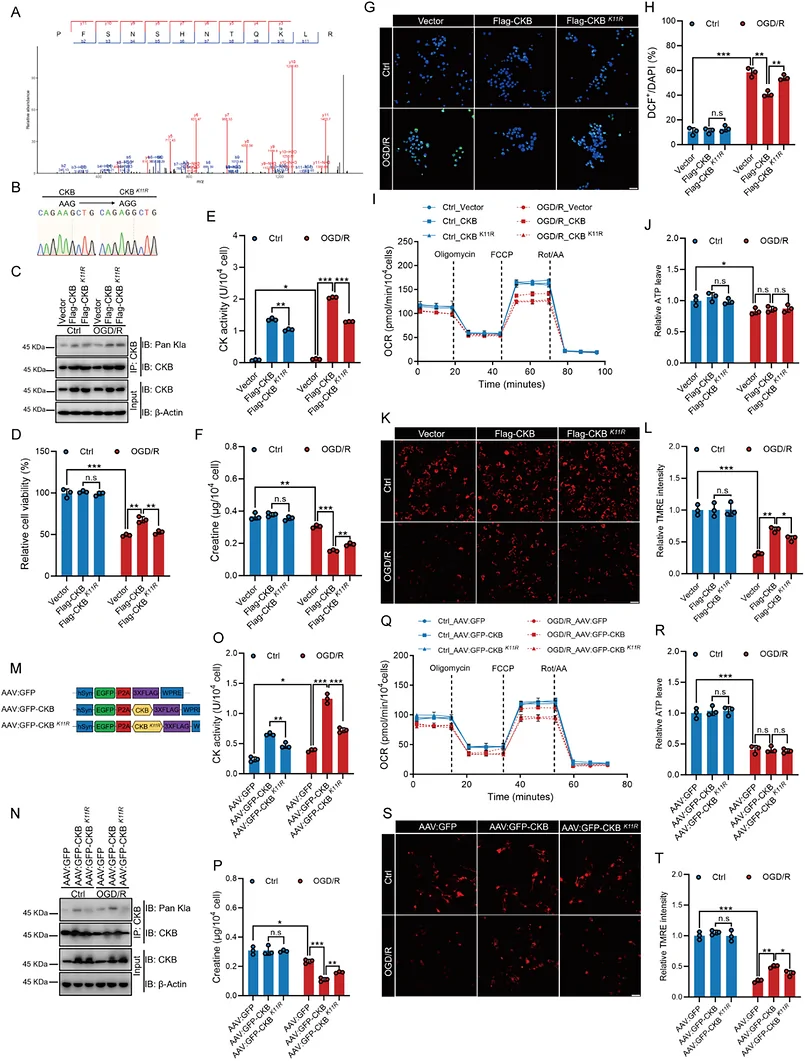
CKB K11 lactylation enhances creatine kinase activity and attenuates OGD/R‐induced oxidative stress. (A) Mass spectrometry spectrum identifying K11‐lactylated CKB peptides in cortical tissues of MCAO/R mice. (B) Sanger sequencing chromatograms validating CKB and CKB *^K11R^* constructs. (C) Immunoblot confirming expression and lactylation levels of Flag‐CKB and Flag‐CKB *^K11R^* in Neuro‐2a cells. (D) Quantitative assessment of cell viability following normoxia or OGD/R treatment in Neuro‐2a cells expressing Flag‐CKB or Flag‐CKB *^K11R^*. *n* = 3 per group. (E, F) CK enzymatic activity (E) and creatine levels (F) under normoxia or OGD/R. *n* = 3 per group. (G, H) Representative images (G) and quantification (H) of ROS (DCFH‐DA staining) in control or OGD/R Neuro‐2a cells. *n* = 3 per group. Scale bar: 50 µm. (I) Mitochondrial respiration capacity following OGD/R treatment in Neuro‐2a cells expressing Flag‐CKB or Flag‐CKB *^K11R^*. *n* = 3 per group. (J) The quantification of ATP level following OGD/R in Neuro‐2a cells expressing Flag‐CKB or Flag‐CKB *^K11R^*. *n* = 3 per group. (K, L) Representative images (K) and quantification (L) of mitochondrial membrane potential (TMRE staining) in control or OGD/R Neuro‐2a cells. *n* = 3 per group. Scale bar: 50 µm. (M) Schematic diagram of AAV‐EGFP‐CKB/CKB *^K11R^*. (N) Immunoblot confirming expression and lactylation levels of expressed AAV‐EGFP‐CKB and AAV‐EGFP‐CKB *^K11R^* primary neuron cells under normoxia or OGD/R. (O, P) CK enzymatic activity (O) and creatine levels (P) of expressed AAV‐EGFP‐CKB and AAV‐EGFP‐CKB *^K11R^* primary neuron cells under normoxia or OGD/R. *n* = 3 per group. (Q) Mitochondrial respiration capacity following OGD/R treatment in expressed AAV‐EGFP‐CKB and AAV‐EGFP‐CKB *^K11R^* primary neuron cells. *n* = 3 per group. (R) The quantification of ATP level following OGD/R in expressed AAV‐EGFP‐CKB and AAV‐EGFP‐CKB *^K11R^* primary neuron cells. *n* = 3 per group. (S, T) Representative images (S) and quantification (T) of mitochondrial membrane potential (TMRE staining) in expressed AAV‐EGFP‐CKB and AAV‐EGFP‐CKB *^K11R^* primary neuron cells under normoxia or OGD/R. *n* = 3 per group. Scale bar: 50 µm. Data are presented as mean ± SEM. Statistical significance was determined by one‐way ANOVA followed by Tukey's post hoc test. ^*^ *p* < 0.05, ^**^ *p* < 0.01, ^***^ *p* < 0.001.

Given the critical contribution of mitochondrial dysfunction and oxidative stress to ischemic injury, we assessed oxygen consumption rate (OCR) using a mitochondrial stress test in Neuro‐2a cells expressing Flag‐CKB or Flag‐CKB *^K11R^* following OGD/R. Consistent with mitochondrial impairment, basal OCR was reduced in all OGD/R‐treated groups compared with control cells. Notably, cells expressing Flag‐CKB exhibited a significantly higher spare respiratory capacity, whereas this adaptive respiratory reserve was largely abolished in cells expressing Flag‐CKB *^K11R^* (Figure 3I). These findings suggest that OGD/R‐induced CKB K11 lactylation enhances mitochondrial respiratory flexibility under stress conditions, despite the absence of a significant change in intracellular ATP levels after OGD/R (Figure 3J). To further delineate the mitochondrial functional consequences of CKB K11 lactylation, we next assessed mitochondrial membrane potential with TMRE staining. OGD/R treatment led to a pronounced reduction in TMRE fluorescence intensity, indicative of mitochondrial depolarization. This decrease was significantly attenuated by Flag‐CKB overexpression, whereas expression of the Flag‐CKB *^K11R^* failed to confer comparable protection (Figure 3K,L). To further validate our findings, we constructed AAVs (adeno‐associated virus) expressing EGFP‐CKB‐Flag and EGFP‐CKB *^K11R^*‐Flag (Figure 3M). Using primary neuron cells as the in vitro model, we transduced these cells with the prepared AAVs and subsequently subjected them to oxygen‐glucose deprivation and reoxygenation (OGD/R) to mimic cerebral ischemia/reperfusion (I/R) injury in vitro. IP combined with western blot assays verified equivalent expression of AAV‐delivered EGFP‐CKB‐Flag and EGFP‐CKB *^K11R^*‐Flag in primary neuron cells. In EGFP‐CKB‐Flag‐expressing neuron cells, OGD/R treatment upregulated CKB K11 lactylation (Figure 3N), along with elevated CK activity and decreased creatine content (Figure 3O,P). In contrast, the K11R mutation largely abolished these OGD/R‐induced metabolic alterations.

We next explored the regulatory role of CKB K11 lactylation in primary neuron cell energy metabolism and mitochondrial function. Seahorse assays demonstrated that OGD/R consistently suppressed energy metabolism across all experimental groups, which was attributed to OGD/R‐mediated oxidative stress. Of note, OGD/R‐challenged primary neuron cells overexpressing EGFP‐CKB‐Flag displayed higher spare respiratory capacity relative to control and EGFP‐CKB *^K11R^*‐Flag groups, which was consistent with our observations in Neuro‐2a cells, despite unaltered cellular ATP levels among groups (Figure 3Q,R). TMRE staining showed that OGD/R exposure significantly dissipated mitochondrial membrane potential in primary neurons. Importantly, EGFP‐CKB‐Flag overexpression partially rescued OGD/R‐induced mitochondrial depolarization (Figure 3S,T).

Collectively, these findings demonstrate that lactylation of CKB at K11 enhances its enzymatic activity under ischemic stress, promotes phosphocreatine‐mediated energy buffering, and mitigates oxidative damage. This modification represents a novel regulatory mechanism contributing to cellular protection during I/R injury.

### 2.4 CKB S199 Phosphorylation Enhances its Creatine Kinase Activity and Protects Against OGD/R‐Induced Injury

Quantitative phosphoproteomic profiling revealed a significant upregulation of CKB phosphorylation at Ser164 (S164) and Ser199 (S199) in MCAO/R mice brains (Figure 2K), corroborated by mass spectrometry data (Figure 4A and Figure S5A). To investigate the functional significance of modification, we constructed these phosphorylation‐deficient mutant plasmids (Flag‐CKB *^S164A^*, Flag‐CKB *^S164D^*, Flag‐CKB *^S199A^*), validated by Sanger sequencing (Figure 4B and Figure S5B,C). Due to the lack of CKB S164‐specific phosphorylation antibodies, only CKB S199 phosphorylation level was assessed using a site‐specific antibody on Flag‐IP in Neuro‐2a cells. The results revealed robust S199 phosphorylation in Flag‐CKB‐transfected cells, whereas this phosphorylation was markedly reduced in Flag‐CKB *^S199A^* (Figure 4C), confirming S199 as a bona fide phosphorylation site. Functionally, viability assessment of Neuro‐2a cells indicated that Flag‐CKB *^S199A^* failed to protect against OGD/R‐induced cytotoxicity, in contrast to Flag‐CKB (Figure 4D). Overexpression of Flag‐CKB significantly increased CK activity post‐OGD/R, whereas the Flag‐CKB *^S199A^* attenuated this enhancement (Figure 4E). Metabolic analysis showed that CKB overexpression led to a depletion of intracellular creatine levels, suggesting elevated phosphocreatine synthesis, while CKB *^S199A^* mutation impaired creatine utilization (Figure 4F). In contrast, ROS staining indicated that Flag‐CKB expression effectively attenuated OGD/R‐induced oxidative stress, whereas Flag‐CKB *^S199A^* exacerbated ROS accumulation (Figure 4G,H).

**FIGURE 4:**
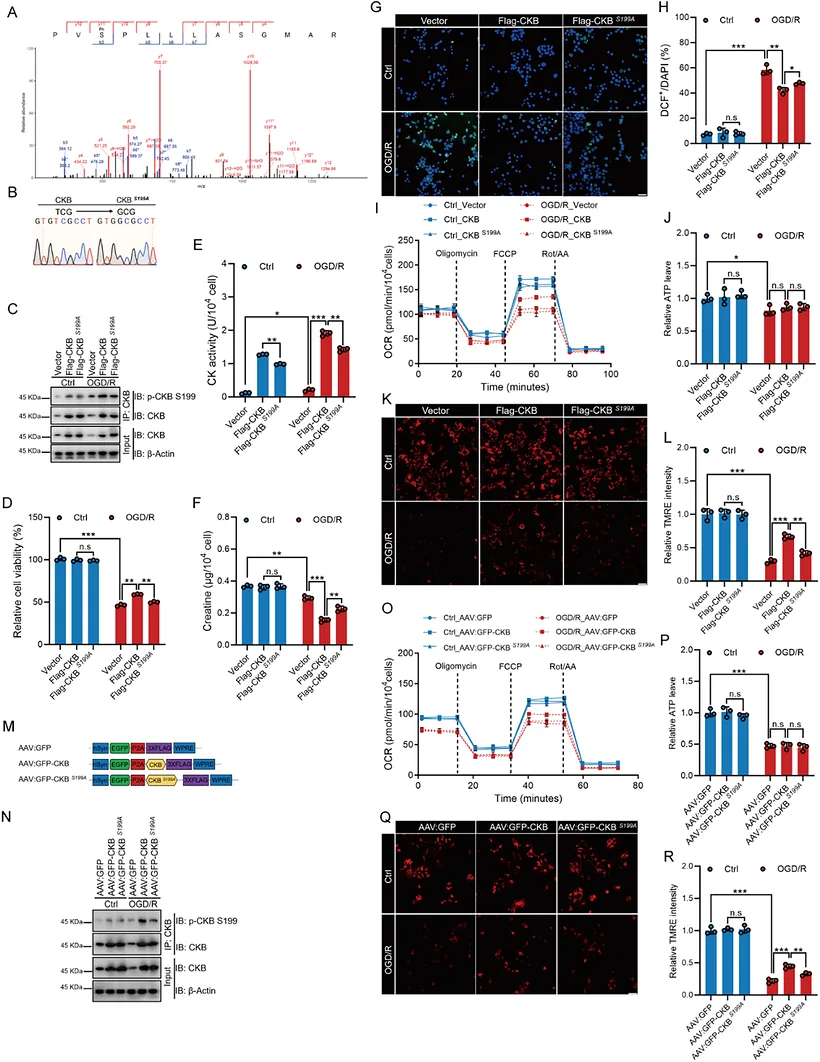
CKB S199 phosphorylation promotes CK activity and protects against OGD/R‐induced oxidative stress. (A) Mass spectrometry spectrum identifying phosphorylated CKB peptides in cortical tissues of MCAO/R mice. (B) Sanger sequencing chromatograms validating CKB and CKB *^S199A^* constructs. (C) Immunoblot confirming expression and phosphorylation levels of Flag‐CKB and Flag‐ CKB *^S199A^* in Neuro‐2a cells. (D) Quantitative assessment of cell viability following OGD/R in Neuro‐2a cells expressing Flag‐CKB or Flag‐CKB *^S199A^*. *n* = 3 per group. (E, F) CK enzymatic activity (E) and creatine levels (F) under normoxia or OGD/R. *n* = 3 per group. (G‐H) Representative images (G) and quantification (H) of ROS (DCFH‐DA staining) in control or OGD/R Neuro‐2a cells. *n* = 3 per group. Scale bar: 50 µm. (I) Mitochondrial respiration capacity following OGD/R treatment in Neuro‐2a cells expressing Flag‐CKB or Flag‐CKB *^S199A^*. *n* = 3 per group. (J) The quantification of ATP level following OGD/R in Neuro‐2a cells expressing Flag‐CKB or Flag‐CKB*^S199A^*. *n* = 3 per group. (K, L) Representative images (K) and quantification (L) of mitochondrial membrane potential (TMRE staining) in control or OGD/R Neuro‐2a cells. *n* = 3 per group. Scale bar: 50 µm. (M) Schematic diagram of AAV‐EGFP‐CKB/CKB *^S199A^*. (N) Immunoblot confirming expression and lactylation levels of expressed AAV‐EGFP‐CKB and AAV‐EGFP‐CKB *^S199A^* primary neuron cells under normoxia or OGD/R. (O) Mitochondrial respiration capacity following OGD/R treatment in expressed AAV‐EGFP‐CKB and AAV‐EGFP‐CKB *^S199A^* primary neuron cells. *n* = 3 per group. (P) The quantification of ATP level following OGD/R in expressed AAV‐EGFP‐CKB and AAV‐EGFP‐CKB *^S199A^* primary neuron cells. *n* = 3 per group. (Q, R) Representative images (Q) and quantification (R) of mitochondrial membrane potential (TMRE staining) in expressed AAV‐EGFP‐CKB and AAV‐EGFP‐CKB *^S199A^* primary neuron cells under normoxia or OGD/R. *n* = 3 per group. Scale bar: 50 µm. Data are presented as mean ± SEM. Statistical significance was determined by one‐way ANOVA followed by Tukey's post hoc test. ^*^ *p* < 0.05, ^**^ *p* < 0.01, ^***^ *p* < 0.001.

Similarly, the functional relevance of CKB S164 phosphorylation was examined by assessing cell viability, CK enzymatic activity, and creatine levels in Neuro‐2a cells expressing Flag‐CKB, Flag‐CKB *^S164A^*, or Flag‐CKB *^S164D^* following OGD/R treatment. The results showed that neither the phosphorylation‐deficient mutant (S164A) nor the phosphomimetic mutant (S164D) produced significant changes in cell viability, CK activity, or creatine content compared with cells expressing wild‐type Flag‐CKB (Figure S5D–I). We further evaluated oxidative stress by measuring intracellular ROS and MDA levels. As shown in Figure S5J–O, ROS and MDA levels were comparable among Neuro‐2a cells expressing Flag‐CKB, Flag‐CKB *^S164A^*, or Flag‐CKB *^S164D^* following OGD/R treatment, indicating no appreciable impact of S164 phosphorylation on oxidative stress responses. The pan Kla of CKB was examined in Neuro‐2a cells expressing Flag‐CKB, Flag‐CKB *^S164A^*, or Flag‐CKB *^S164D^* following OGD/R treatment. The results showed that neither the Flag‐CKB *^S164D^* nor the Flag‐CKB *^S164A^* produced significant changes in CKB pan Kla expression (Figure S5P,Q). Collectively, these results suggest that phosphorylation at CKB S164 has no significant influence on CKB enzymatic activity, cellular metabolism, or oxidative stress under OGD/R conditions, thereby supporting the selective focus on S199 phosphorylation in subsequent mechanistic analyses.

We next investigated whether CKB S199 phosphorylation similarly influences mitochondrial function. Consistent with the observations in Figure 3, OCR analysis demonstrated a general reduction in mitochondrial respiration following OGD/R, while cells expressing Flag‐CKB displayed a selectively increased spare respiratory capacity. This adaptive response was absent in cells expressing Flag‐CKB *^S199A^* (Figure 4I). Finally, intracellular ATP and ROS levels were measured. ATP levels remained comparable among all OGD/R‐treated groups (Figure 4J). TMRE staining revealed that OGD/R‐induced mitochondrial depolarization was partially rescued by Flag‐CKB overexpression, whereas Flag‐CKB S199A showed a significantly weaker protective effect (Figure 4K,L).

Likewise, primary neuron cells were transduced with the constructed AAV vectors (Figure 4M) and subsequently subjected to oxygen–glucose deprivation/reoxygenation (OGD/R) treatment to establish an in vitro model of cerebral ischemia/reperfusion (I/R) injury. IP coupled with western blot analysis confirmed equivalent expression of AAV‐mediated EGFP‐CKB‐Flag and EGFP‐CKB *^S199A^*‐Flag in primary neuron cells. In primary neurons overexpressing EGFP‐CKB‐Flag, OGD/R stimulation induced the upregulation of CKB S199 phosphorylation, but in EGFP‐CKB *^S199A^*‐Flag in primary neuron cells (Figure 4N). We further interrogated the regulatory function of CKB S199 phosphorylation in modulating energy metabolism and mitochondrial homeostasis in primary neurons. Seahorse extracellular flux assays revealed that OGD/R injury uniformly repressed cellular energy metabolism in all OGD/R groups, which was attributable to OGD/R induced oxidative stress. Intriguingly, OGD/R‐stimulated primary neurons with EGFP‐CKB‐Flag overexpression presented elevated spare respiratory capacity compared with control and EGFP‐CKB *^S199A^*‐Flag groups. This phenotypic pattern was consistent with our previous results in Neuro‐2a cells, while no significant differences in cellular ATP levels were detected across groups (Figure 4O,P). TMRE staining assays validated that OGD/R exposure led to a pronounced dissipation of mitochondrial membrane potential in primary neuron cells. Notably, primary neuron cells expressing of EGFP‐CKB‐Flag effectively ameliorated OGD/R‐evoked mitochondrial depolarization compare to EGFP‐CKB *^S199A^*‐Flag, exerting a partial rescuing effect on mitochondrial dysfunction (Figure 4Q,R).

These results demonstrate that CKB S199 phosphorylation enhances CKB enzymatic activity, facilitates creatine metabolism, augments mitochondrial spare respiratory capacity, and attenuates oxidative damage, thereby contributing to cytoprotection under I/R stress.

### 2.5 GCN5 is the Acyltransferase for CKB K11 lactylation, and Sirt5 Functions as its Delactylase

Given the pivotal role of CKB K11 lactylation in I/R tolerance, we sought to identify the regulatory enzymes responsible for its dynamic modulation. We screened representative histone acetyltransferases (HATs)‐of P300, CBP, GCN5, and PCAF‐using co‐IP assays with Flag‐CKB and HA‐tagged constructs. Among these, both HA‐GCN5 and HA‐PCAF showed enrichment, but only GCN5 overexpression significantly increased CKB lactylation levels (Figure 5A). PCAF also showed interaction but did not significantly alter CKB lactylation, thus excluded from further analysis. In vitro Co‐IP further confirmed the interaction between GCN5 and CKB (Figure 5B,C). Functionally, GCN5 overexpression enhanced CKB K11 lactylation and promoted cell viability following OGD/R injury (Figure 5D). When co‐transfected with Flag‐CKB, GCN5 further boosted CK enzymatic activity and decreased intracellular creatine levels in Neuro‐2a cells under OGD/R conditions compared to CKB expression alone (Figure 5E,F), indicating that GCN5 potentiates CK activity through enhanced CKB lactylation. To further assess the involvement of GCN5 in regulating CKB K11 lactylation, we pharmacologically inhibited GCN5 activity using the small‐molecule inhibitor MB‐3 and examined its impact on CKB lactylation and function. As shown in Figure 5G, MB‐3 treatment significantly reduced OGD/R‐induced CKB lactylation without affecting the GCN5–CKB interaction, while the GCN5‐CKB interaction remained largely unchanged, which is consistent with the mode of action of enzymatic activity inhibition rather than disruption of protein‐protein association. We further assessed endogenous CKB K11 lactylation via CKB IP in MB‐3‐treated Neuro‐2a cells. Western blot results demonstrated that OGD/R markedly enhanced CKB K11 lactylation compared with the Ctrl group, whereas MB‐3 treatment significantly suppressed OGD/R‐induced CKB K11 lactylation by inhibiting GCN5 activity (Figure 5H). Moreover, quantitative detection of CK activity, creatine, and cellular ATP levels revealed that MB‐3 treatment substantially reduced CK enzymatic activity and elevated intracellular creatine abundance following OGD/R injury, with no significant alterations in cellular ATP levels (Figure 5I–K).

**FIGURE 5:**
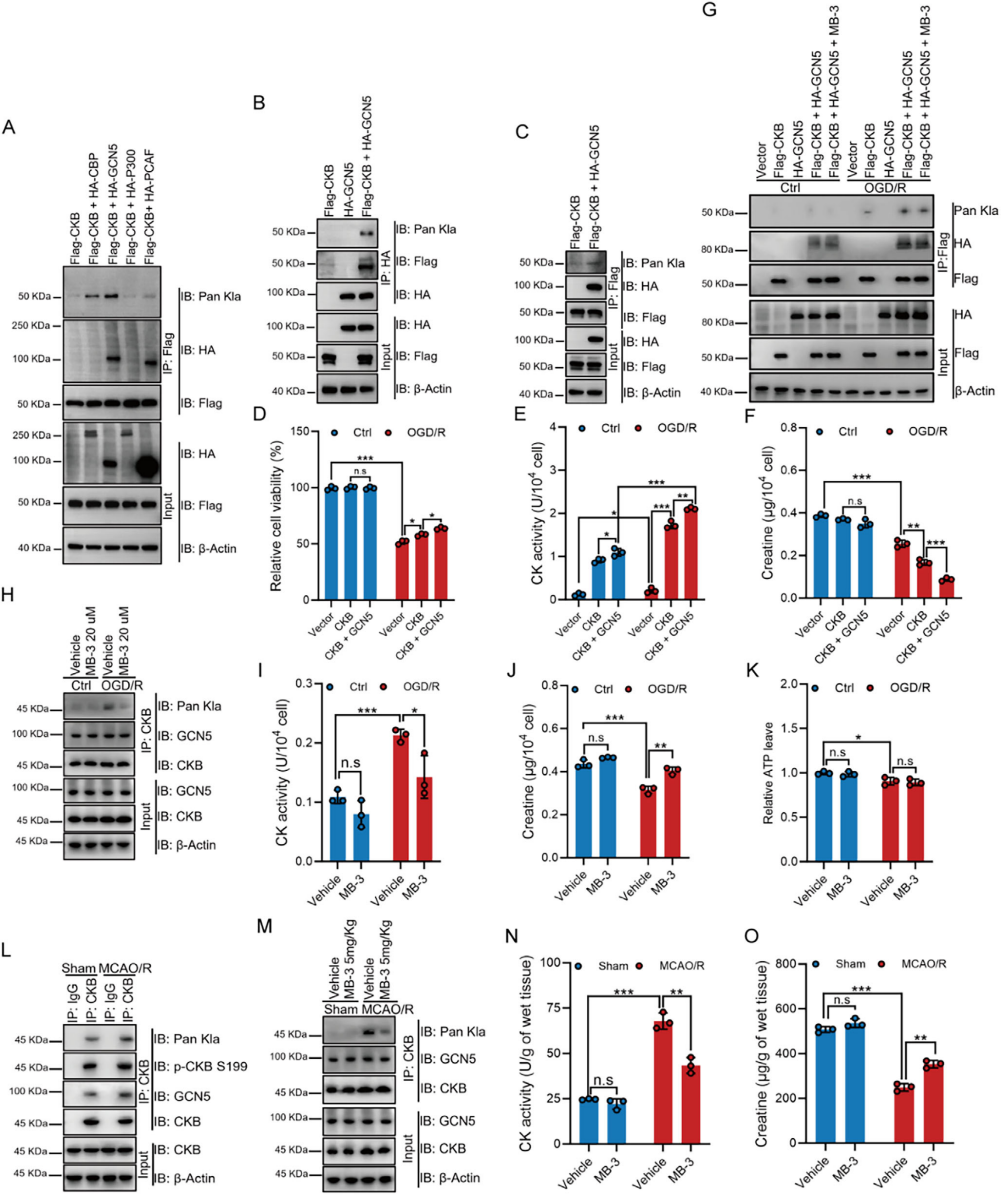
GCN5 functions as the lactyltransferase catalyzing CKB K11 lactylation. (A) Different lactyltransferases were screened by IP assays. Flag‐CKB was co‐expressed with HA‐tagged CBP, p300, PCAF, or GCN5 in Neuro‐2a cells; lysates were subjected to IP and immunoblot for Pan‐Kla, HA, and Flag. (B‐C) IP assays showing GCN5‐dependent lactylation of CKB. Flag‐CKB or Flag‐CKB + HA‐GCN5 were expressed in Neuro‐2a cells (B); Flag‐CKB, HA‐GCN5, or Flag‐CKB + HA‐GCN5 were expressed in Neuro‐2a cells (C); lysates were subjected to IP and immunoblot for Pan‐Kla, HA, and Flag. (D–F) Neuro‐2a cells were transfected with Flag‐CKB or Flag‐CKB + HA‐GCN5 and subjected to normoxia or OGD/R; Cell viability (D), CK activity (E), and creatine levels (F) were examined and quantified. *n* = 3 per group. (G) IP assay to verify the function of GCN5 as a CKB K11 lactyltransferases. Flag‐CKB was co‐expressed with HA‐GCN5 in Neuro‐2a cells and treated with or without MB‐3 following OGD/R; lysates were subjected to IP and immunoblot for Pan‐Kla, HA, and Flag. (H) Neuro‐2a cells were treated with or without MB‐3 following OGD/R treatment; lysates were subjected to CKB‐IP and immunoblot for Pan‐Kla, GCN5, and CKB. (I–K) Neuro‐2a cells were treated with or without MB‐3 following OGD/R, CK activity (I), creatine levels (J), and ATP levels (K) were examined and quantified. *n* = 3 per group. (L) Mice cerebral cortex tissues were fragmented and digested after MCAOR treatment, lysates were subjected to CKB‐IP and immunoblot for Pan‐Kla, p‐CKB S199, GCN5, and CKB. (M) Mice were intraperitoneally injected with 5mg/kg MB‐3 or vehicle for 5 days before MCAO/R modeling, and the cerebral cortex tissues of the mice following MCAO/R were digested. Lysates were subjected to CKB‐IP and immunoblot for Pan‐Kla, GCN5, and CKB. (N, O) Mice were intraperitoneally injected with 5mg/kg MB‐3 or vehicle for 5 days before MCAO/R modeling, and cerebral cortex tissues of the mice following MCAO/R were digested, CK activity (N), creatine levels (O) were examined and quantified. *n* = 3 per group. Data are presented as mean ± SEM. Statistical significance was determined by one‐way ANOVA followed by Tukey's post hoc test. ^*^ *p* < 0.05, ^**^ *p* < 0.01, ^***^ *p* < 0.001.

To corroborate the above in vitro findings, we further performed in vivo validation using MCAO/R model. Mice were intraperitoneally administered with MB‐3 (5 mg/kg) for five consecutive days prior to MCAO/R surgery. IP assays of cortical tissue lysates verified an endogenous interaction between GCN5 and CKB, and this protein‐protein interaction was moderately strengthened upon MCAO/R injury (Figure 5L). Consistent with our in vitro results, CKB IP further confirmed that MB‐3 treatment effectively diminished endogenous CKB K11 lactylation in ischemic brain tissues (Figure 5M). Quantification of ischemic cortical samples also demonstrated that MB‐3 intervention significantly inhibited CK activity and restored creatine after MCAO/R (Figure 5N,O). Consistently, these data support a functional role for GCN5 enzymatic activity in modulating CKB K11 lactylation and CKB‐dependent metabolic responses under ischemia‐reperfusion conditions.

To identify potential delactylases, we screened sirtuin family members and found that Sirt5 directly interacts with CKB and reduces its lactylation upon overexpression (Figure S6A–C). Co‐expression of HA‐Sirt5 with Flag‐CKB abolished the CKB‐mediated increase in cell viability following OGD/R (Figure S6D). CK activity assays showed that while CKB overexpression elevated enzymatic activity under both normoxia and OGD/R conditions, Sirt5 overexpression selectively suppressed CK activity under OGD/R stress (Figure S6E,F).

Cumulatively, these data identify GCN5 as the CKB K11 acyltransferase and Sirt5 as its counteracting delactylase, forming a dynamic regulatory axis that modulates CKB activity and I/R neuroprotection.

### 2.6 CKB S199 Phosphorylation Promotes GCN5‐Mediated K11 Lactylation and Enhances Cytoprotection

Both CKB S199 phosphorylation and K11 lactylation enhance CK enzymatic activity and protect against OGD/R‐induced injury. Given the spatial proximity of these two modification sites within the CKB structure (20 amino acids apart), a potential regulatory interplay between these PTMs is suggested. To explore this possibility, we examined whether one modification influences the occurrence of the other under OGD/R conditions. IP analysis in OGD/R‐exposed Neuro‐2a cells showed that while Flag‐CKB overexpression increased Pan‐Kla, this effect was abolished in the lactylation‐deficient mutant Flag‐CKB *^K11R^* without affecting CKB S199 phosphorylation (Figure 6A). In contrast, the phospho‐deficient mutant Flag‐CKB *^S199A^* displayed reduced S199 phosphorylation accompanied by decreased lactylation (Figure 6B), suggesting that CKB K11 lactylation is dependent on its phosphorylation at S199. To further confirm this regulatory relationship, we generated a phosphomimetic mutant (Flag‐CKB *^S199D^*) that mimics constitutive phosphorylation at S199 (Figure 6C). In both control and OGD/R conditions, Flag‐CKB *^S199D^* exhibited elevated CKB phosphorylation and enhanced K11 lactylation compared to Flag‐CKB (Figure 6D), supporting the conclusion that S199 phosphorylation promotes K11 lactylation.

**FIGURE 6:**
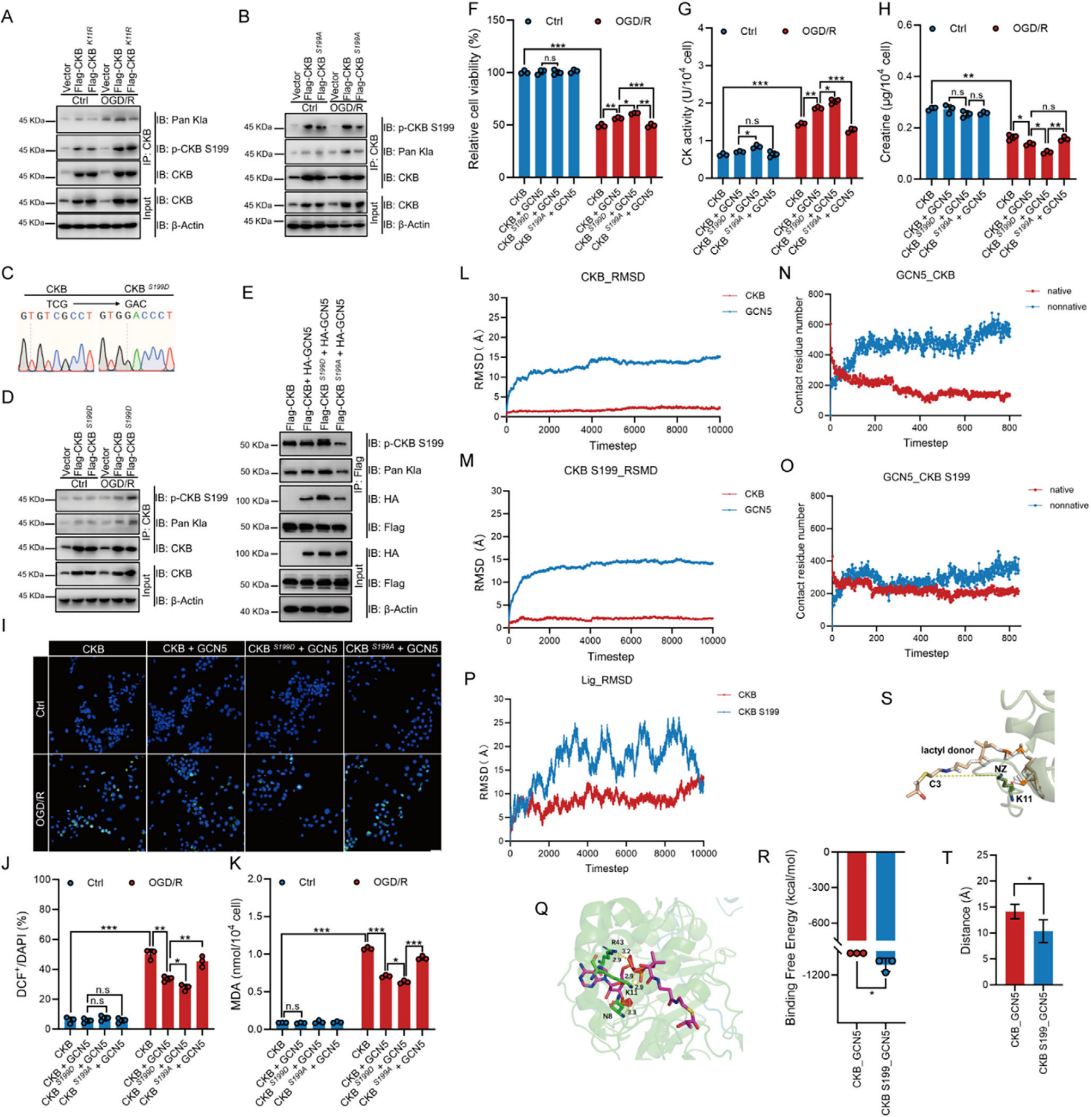
S199 phosphorylation enhances the GCN5‐CKB interaction to promote lactylation and neuroprotection. (A) Pan‐Kla and p‐CKB S199 in Neuro‐2a cells expressing Flag‐CKB or Flag‐CKB *^K11R^* were analyzed by IP assay after Ctrl and OGD/R treatment. (B) Pan‐Kla and p‐CKB S199 in Neuro‐2a cells expressing Flag‐CKB or Flag‐CKB *^S199A^* were analyzed by IP assay after Ctrl and OGD/R treatment. (C) Sequencing confirmation of CKB *^S199D^* phosphomimetic construct. (D) Immunoblots of CKB phosphorylation and lactylation in Flag‐CKB vs. Flag‐CKB *^S199D^* transfected Neuro‐2a cells under control and OGD/R conditions. (E) IP analysis of GCN5‐CKB interactions with different CKB variants. (F–H) CK activity (F), creatine levels (G), and cell viability (H) in Neuro‐2a cells with various transfection under control and OGD/R conditions. *n* = 3 per group. (I, J) Representative images (I) and quantification (J) of ROS staining by DCFH‐DA probe in Neuro‐2a cells with various transfection under control and OGD/R treatment. *n* = 3 per group. Scale bar: 50 µm. (K) The quantification of MDA level following OGD/R in HA‐GCN5 and Flag‐CKB, Flag‐CKB *^S199A^*, or Flag‐CKB *^S199D^* co‐transfected Neuro‐2a cells. *n* = 3 per group. (L, M) RMSD trajectories of GCN5‐CKB docking complexes containing CKB (L) or CKB S199 (M) throughout molecular docking simulations. (N, O) Dynamic variations in interfacial contacts within GCN5‐CKB docking systems of CKB (N) and CKB S199 (O). (P) RMSD fluctuation curves of lactyl donor docked to CKB or CKB S199. (Q) Schematic diagram illustrating hydrogen‐bond interactions between lactyl donor and CKB amino acid residues. (R) Calculated BFE obtained from molecular docking between GCN5 and CKB / CKB S199. (S, T) Schematic diagram (S) and statistical histogram (T) of the average distance between the lactoyl donor–C3 and K11 amino acid residue–NZ in CKB / CKB S199 obtained via molecular docking simulations. Data are presented as mean ± SEM. Statistical significance was determined by one‐way ANOVA followed by Tukey's post hoc test. ^*^ *p* < 0.05, ^**^ *p* < 0.01, ^***^ *p* < 0.001.

Given the role of PTMs in modulating protein–protein interactions, we next examined whether CKB S199 phosphorylation influences binding to its modifying enzymes. Flag IP assays showed that Flag‐CKB *^S199D^* significantly enhanced the interaction with HA‐GCN5 and elevated Pan‐Kla levels, whereas the S199A mutant reduced both (Figure 6E). Functionally, co‐expression of Flag‐CKB *^S199D^* and HA‐GCN5 increased CK activity, decreased cellular creatine levels, and increased cell viability (Figure 6F–H), while reducing oxidative stress markers (MDA, ROS) more effectively than wild‐type CKB (Figure 6I–K). In contrast, Flag‐CKB *^S199A^* impaired these protective effects. These data demonstrate that phosphorylation at S199 enhances GCN5 binding, thereby promoting CKB lactylation and augmenting its enzymatic and cytoprotective functions.

We next performed molecular docking simulations to explore the effects of wild‐type (WT) CKB and phosphorylated S199 CKB (p‐S199 CKB) on the interaction between CKB and GCN5. RMSD analysis (Figure 6L,M) revealed that CKB remained structurally stable in both WT and p‐S199 CKB systems, whereas GCN5 exhibited substantial relative displacement in both groups, indicating evident interfacial rearrangement of the CKB‐GCN5 complex. Interfacial contact analysis (Figure 6N,O) further showed that the WT CKB system displayed a marked reduction in initial contact numbers accompanied by a continuous increase in new contacts, suggesting extensive loss of native interfacial interactions and prominent structural rearrangement of the CKB‐GCN5 complex. In contrast, the p‐S199 CKB system retained more initial interfacial contacts with a milder increase in newly formed contacts, demonstrating that S199 phosphorylation facilitates the maintenance of the native binding mode of the CKB‐GCN5 complex.

RMSD analysis of the lactyl donor (Figure 6P) showed that the lactyl donor exhibited a higher RMSD value in the p‐S199 CKB system relative to CKB system, indicative of a distinct reorientation of its binding conformation. Furthermore, binding free energy (BFE) analysis revealed a lower binding energy between GCN5 and CKB in the p‐S199 CKB system (Figure 6Q,R), along with a significant reduction in the average distance between the lactyl donor C3 and CKB K11 NZ atoms (Figure 6S,T). Collectively, these findings suggest that CKB S199 phosphorylation stabilizes the local CKB‐GCN5 interface, thereby providing a favorable and stable conformational basis for GCN5‐mediated lactylation at the CKB K11 site.

To assess whether S199 phosphorylation also affects the interaction with the delactylase Sirt5, we performed co‐transfections with HA‐Sirt5. Unlike GCN5, Sirt5 binding to CKB was unaffected by the phosphorylation status (CKB, CKB *^S199A^* or CKB *^S199D^*), and Sirt5 overexpression did not significantly alter CK activity, viability, or oxidative stress in Neuro‐2a cells overexpressing S199A, or S199D mutant CKB, compared to CKB (Figure S7A–G). These findings suggest that CKB S199 phosphorylation selectively promotes GCN5‐dependent lactylation without interfering with Sirt5‐mediated delactylation.

### 2.7 Dual Post‐Translational Modifications of CKB K11 lactylation and S199 Phosphorylation Jointly Confer Neuroprotection Against Cerebral I/R injury in Mice

Given that enhanced K11 lactylation, S199 phosphorylation, and CKB activity were observed in OGD/R‐treated neurons, we next examined whether these 2 modifications similarly occur in the brain following MCAO/R and contribute to functional recovery through the same enzymatic activation mechanism. We constructed AAV vectors encoding wild‐type CKB (AAV_CKB) or site‐specific mutants (K11R and S199A), driven by the neuron‐specific hSyn promoter, and administered them via tail vein injection. Following a 3‐week expression period to allow sufficient transduction, mice were subjected to MCAO/R surgery, and both acute (24 h) and chronic (4 weeks) outcomes were evaluated in accordance with the experimental timeline (Figure 7A). No significant difference in AAV transduction efficiency was detected across all groups prior to MCAO/R induction (Figure 7B,C). IP assays confirmed that MCAO/R robustly induced both CKB K11 lactylation and S199 phosphorylation, and these modifications were markedly reduced in the respective mutant groups, validating successful neuronal transduction and specific disruption of the target sites (Figure 7D,E).

**FIGURE 7:**
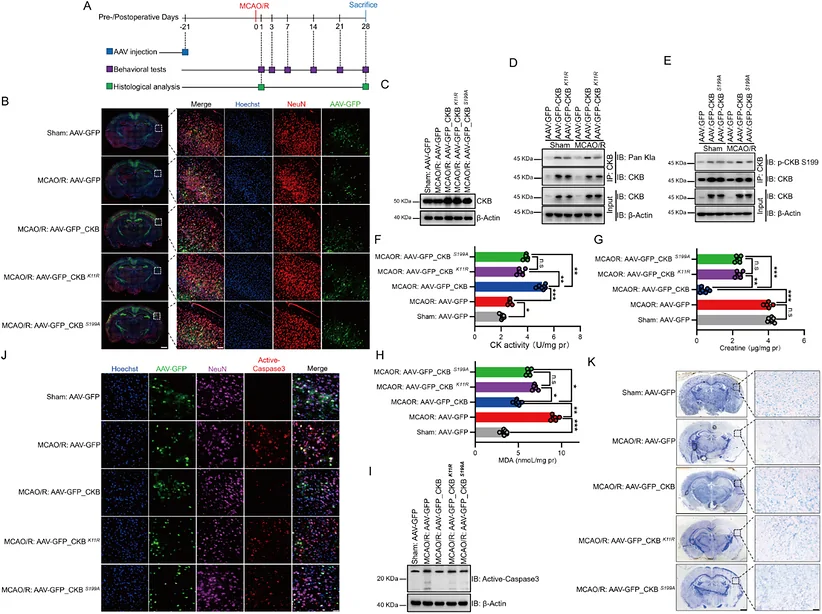
CKB K11 lactylation and S199 phosphorylation protect against cerebral I/R injury in vivo. (A) Timeline of in vivo experimental procedures. (B) Immunofluorescence showing GFP expression in cortex 3 weeks post‐AAV injection. Scale bar: 100 µm. (C) Western blots detecting CKB expression in cortex 3 weeks post‐AAV injection. (D, E) CKB‐IP combined with Western blots detecting K11 lactylation (D) and S199 phosphorylation (E) post‐MCAO/R. (F, G) CK activity (F) and creatine levels (G) in each group. (*n* = 5 per group). (H) MDA levels in cortical lysates of each group. (*n* = 5 per group). (I, J) Detection of active‐Caspase‐3 in mice cortex across groups by (I) Western blot and (J) immunofluorescence staining. Scale bar: 50 µm. (K) Representative Nissl‐stained sections showing neuronal morphology and Nissl body density in cortical tissues at 24 h after MCAO/R. Scale bar: 200 µm. Data are presented as mean ± SEM. Statistical significance was determined by one‐way ANOVA followed by Tukey's post hoc test. ^*^ *p* < 0.05, ^**^ *p* < 0.01, ^***^ *p* < 0.001.

Consistent with in vitro findings, cortical CK activity and creatine levels were significantly reduced in mice expressing the K11R or S199A mutants compared to those expressing wild‐type CKB following MCAO/R (Figure 7F,G), supporting the role of these PTMs in enzymatic activation. At the oxidative stress level, AAV_CKB‐transduced mice exhibited significantly lower MDA accumulation relative to the AAV‐GFP control group, whereas this antioxidative effect was partially lost in both mutant groups (Figure 7H).

We next evaluated the anti‐apoptotic effects of CKB PTMs. Western blot analysis revealed a substantial reduction in Active‐Caspase‐3 expression in the AAV‐GFP_CKB group, whereas AAV‐GFP_CKB *^K11R^* and AAV‐GFP_CKB *^S199A^* mice showed only modest reductions (Figure 7I). These results were further supported by immunofluorescence staining, which demonstrated a significant decrease in Active‐Caspase‐3‐positive cells in AAV‐GFP_CKB‐expressing mice, but not in those expressing the mutant constructs (Figure 7J). Histopathological examination by Nissl staining revealed extensive neuronal damage, characterized by shrunken Nissl bodies and disrupted cortical cytoarchitecture, in the AAV‐GFP group. In contrast, mice expressing wild‐type CKB exhibited preserved neuronal morphology, enlarged nissl bodies, and reduced neuronal loss. However, these neuroprotective features were only partially retained in AAV‐GFP_CKB *^K11R^* and AAV‐GFP_CKB *^S199A^* groups (Figure 7K).

Together, these findings demonstrate that both K11 lactylation and S199 phosphorylation are critical for full activation of CKB's kinase activity and its neuroprotective functions. The loss of either modification compromises CKB‐mediated protection against oxidative stress, neuronal apoptosis, and histological damage following ischemic insult, underscoring the synergistic contribution of these PTMs in cerebral I/R injury.

### 2.8 CKB K11 Lactylation and S199 Phosphorylation Synergistically Preserve Brain Integrity and Promote Long‐Term Functional Recovery Following Cerebral I/R Injury in Mice

To comprehensively evaluate the neuroprotective effects of CKB K11 lactylation and S199 phosphorylation following cerebral ischemia/reperfusion (I/R) injury, we assessed both structural and functional outcomes in MCAO/R mice treated with AAV‐GFP, AAV‐GFP_CKB, AAV‐GFP_CKB *^K11R^*, or AAV‐GFP_CKB *^S199A^*. Our analysis focused on infarct volume, motor behavior, neuronal survival, and long‐term brain atrophy. TTC staining revealed that mice receiving AAV‐GFP_CKB exhibited a significantly reduced infarct percentage compared with the AAV_GFP control group. In contrast, this reduction was markedly attenuated in the AAV‐GFP_CKB *^K11R^* and AAV‐GFP_CKB *^S199A^* groups, indicating a diminished neuroprotective effect when K11 lactylation or S199 phosphorylation was disrupted (Figure 8A,B).

**FIGURE 8:**
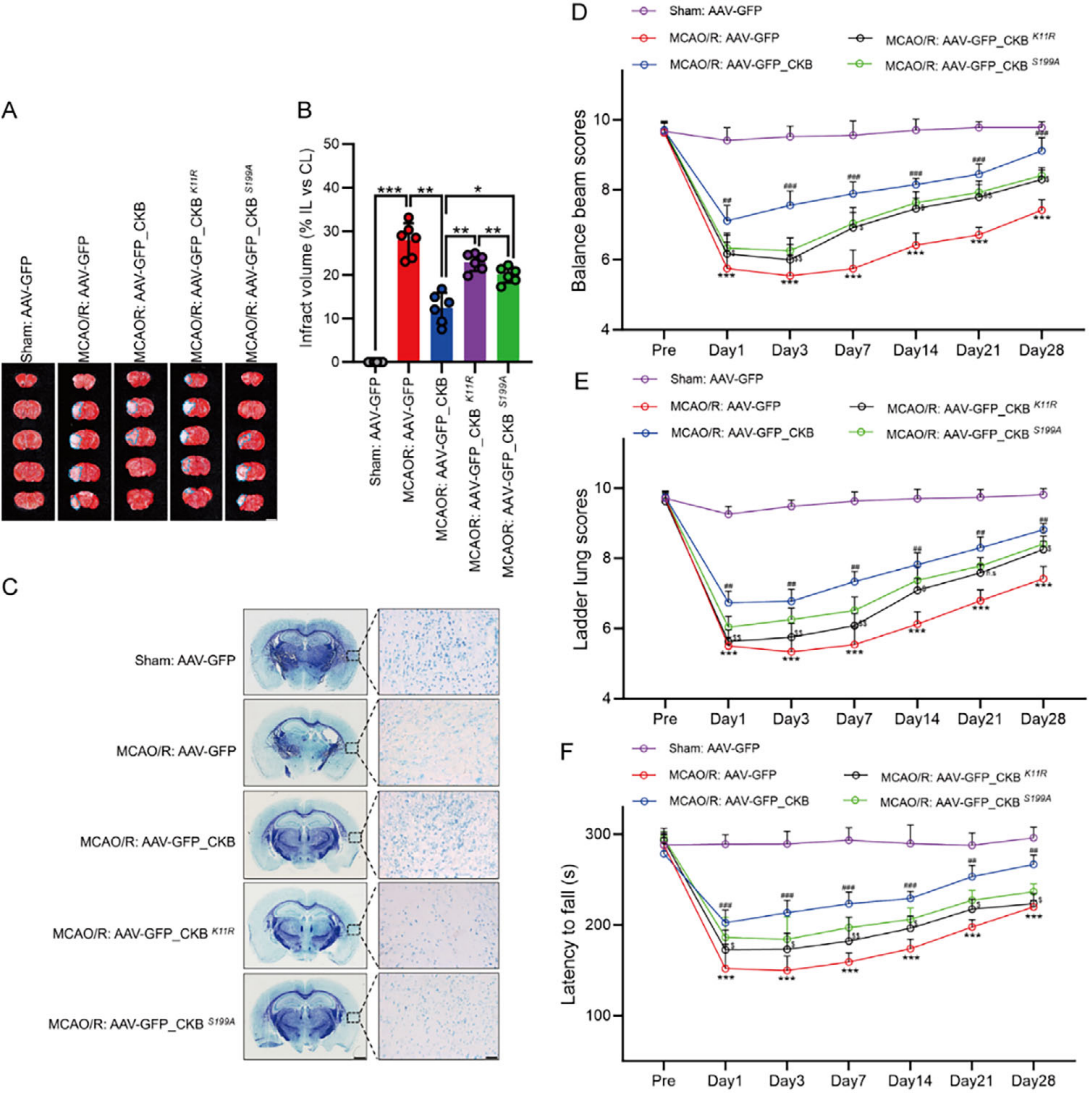
CKB K11 lactylation and S199 phosphorylation protect against neuronal injury and promote long‐term functional recovery following cerebral I/R in mice. (A) Representative TTC‐stained brain sections from mice 24 h after MCAO/R surgery. Infarct areas appear pale, while viable tissue stains red. Scale bar: 2 mm. (B) Quantification of infarct volume expressed as a percentage of the ipsilateral hemisphere across groups. (*n* = 6 per group). (C) Representative nissl‐stained sections showing neuronal morphology and Nissl body density in cortical tissues at 28 days after MCAO/R. AAV_CKB‐treated mice displayed preserved neuronal integrity compared to control and mutant groups. Scale bar: 200 µm. (D–F) Longitudinal evaluation of motor function recovery using balance beam test (D), ladder rung walking test (E), and rotarod performance test (F) at various timepoints. (*n* = 9 per group). Data are presented as mean ± SEM. Statistical significance was determined by one‐way ANOVA followed by Tukey's post hoc test. ^*^ *p* < 0.05, ^**^ *p* < 0.01, ^***^ *p* < 0.001.

Histological analysis using Nissl staining at 4 weeks post‐MCAO/R showed severe neuronal loss and morphological disruption in the AAV_GFP group, which was significantly ameliorated in AAV‐GFP_CKB‐treated mice. Restoration of Nissl body volume and cytoarchitecture was most robust in the wild‐type group, while partial improvements were observed in the AAV‐GFP_CKB *^K11R^* and AAV‐GFP_CKB *^S199A^* groups (Figure 8C).

To investigate functional outcomes, we performed a battery of behavioral tests, including balance beam, ladder rung, and rotarod over a four‐week post‐surgical period. MCAO/R induced pronounced motor deficits in AAV_GFP‐treated mice relative to sham controls (Figure 8D–F). Notably, mice expressing AAV‐GFP_CKB demonstrated significant improvement in motor coordination and balance throughout the observation period. In contrast, mice expressing the AAV‐GFP_CKB *^K11R^* and AAV‐GFP_CKB *^S199A^* exhibited only modest recovery, further supporting the necessity of these PTMs for functional restoration.

Collectively, these data indicate that CKB‐mediated K11 lactylation and S199 phosphorylation are essential not only for acute neuroprotection but also for sustained recovery of brain structure and motor function after I/R insult. Enhancing CKB enzymatic activity through these modifications may represent a critical endogenous mechanism to counteract I/R‐ induced energy failure, oxidative stress, and neuronal degeneration.

## 3 Discussion

To systematically profile post‐translational modifications associated with cerebral ischemia‐reperfusion (I/R) injury, we conducted an integrated analysis combining proteomic, lactylomic, and phosphoproteomic profiling in a murine MCAO/R model. This comprehensive approach revealed widespread alterations in lysine lactylation, particularly affecting proteins involved in synaptic transmission, excitatory signaling, and neuronal calcium homeostasis [42, 43]. These findings suggest that lysine lactylation, beyond being a metabolic byproduct, may participate in active modulation of neuronal responses to ischemic stress. Notably, among the differentially modified proteins, we identified a novel PTM of CKB K11 lactylation that enhances enzymatic activity and confers neuroprotection in the context of cerebral I/R injury. This modification is significantly upregulated following I/R and closely regulated by the lysine acetyltransferase GCN5. Functionally, CKB K11 lactylation increase mitochondria spare respiratory capacity and reduces neuronal apoptosis, underscoring its importance in sustaining cellular bioenergetics under ischemic stress. In parallel, we provide, to our knowledge, the first evidence that CKB S199 phosphorylation facilitates enzymatic activity and exerts a synergistic protective effect, consistent with the site's prior identification in 2010 [44]. Intriguingly, S199 phosphorylation enhances CKB K11 lactylation, suggesting a coordinated interplay between phosphorylation and lactylation in modulating CKB function (Figure S8).

Lactate plays a dual role in cerebral ischemic injury. On one hand, accumulating evidence suggests that exogenous lactate can serve as an alternative energy substrate and exert neuroprotective effects under hypoxic‐ischemic conditions [20, 21]. On the other hand, excessive endogenous lactate accumulation during acute ischemia is a well‐recognized contributor to injury progression, primarily through exacerbating intracellular acidosis and mitochondrial dysfunction [22, 23, 24]. Our findings refine this apparent paradox by demonstrating that the impact of lactate is not determined solely by its abundance, but by how it is metabolically and post‐translationally utilized. Specifically, we show that lactate‐derived K11 lactylation of CKB, facilitated by S199 phosphorylation, enhances creatine kinase activity and supports energy buffering under ischemia‐reperfusion stress. This regulated utilization of lactate contrasts with nonspecific lactate accumulation, which is more likely to exacerbate cellular injury. Together, these results support a context‐dependent model in which lactate contributes to neuroprotection when properly integrated into PTM‐mediated adaptive pathways, but becomes detrimental when dysregulated, highlighting the importance of lactate metabolic fate rather than lactate levels alone.

The spatial proximity between S199 and K11 within the CKB protein structure implies that phosphorylation‐induced conformational changes may facilitate access of lactyltransferases to K11. GCN5 was confirmed as a key lactyltransferase mediating K11 modification; however, the phosphorylation event at S199 appeared to further amplify lactylation levels independent of changes in GCN5 expression, pointing toward a structural or recruitment‐based mechanism. This crosstalk between phosphorylation and lactylation reveals a layer of PTM coordination that fine‐tunes the enzymatic activity of metabolic proteins during neuronal stress responses [45].

Importantly, our in vivo experiments showed that mice expressing CKB harboring the K11R or S199A mutation exhibited worsened neurological outcomes and increased infarct volumes following ischemia‐reperfusion injury, compared with mice expressing wild‐type CKB. In contrast, wild‐type CKB overexpression was associated with reduced tissue damage and improved neurological performance. Nevertheless, we acknowledge that the relatively limited sample size in the present in vivo experiments represents a constraint on the precise estimation of the observed effects. Future studies incorporating larger cohorts will therefore be valuable for further validating the observed effects on infarct volume and neurological outcomes. These in vivo findings, in conjunction with our mechanistic analyses, support the physiological relevance of CKB lactylation and phosphorylation in modulating ischemic outcomes [46, 47, 48]. Moreover, the reprogramming of lactate metabolism, at least in part mediated by enhanced CKB activity, may represent a compensatory mechanism to buffer energy crisis in the ischemia‐reperfusion brain, thereby supporting a protective role of PTM‐regulated creatine cycling.

From a translational perspective, the dual modulation of CKB by K11 lactylation and S199 phosphorylation offers promising therapeutic implications. Strategies aimed at promoting both modifications, via metabolic reprogramming [49], lactate signaling modulation, or kinase/phosphatase targeting, may synergistically restore energy balance and neuronal survival after stroke [43, 50]. In particular, the interdependence of these PTMs suggests that targeting one modification could influence the other, offering a means of fine‐tuning CKB activity in a context‐dependent manner.

Additionally, our findings may have relevance beyond acute ischemia. Impaired CK function has been implicated in neurodegenerative diseases, including Alzheimer's and Parkinson's, where energy dysregulation is a common feature [51, 52]. It remains to be explored whether dysregulated PTMs of CKB contribute to chronic neurodegeneration and whether restoring K11 lactylation and S199 phosphorylation could confer long‐term neuroprotection.

Despite the promising findings, several limitations warrant consideration. Given the technical limitations of genetic manipulation in primary neurons and Neuro‐2a cells, exogenous overexpression of different CKB mutants via plasmid transfection and AAV transduction was adopted for in vitro functional assays. Future investigation will focus on establishing endogenous mutation neuron cell models and validating the underlying mechanisms in intact in vivo systems, which will further deepen and complement our current mechanistic understanding. Regarding the molecular mechanism, although our data suggest a regulatory interplay between S199 phosphorylation and K11 lactylation, the precise mechanism, particularly how phosphorylation facilitates lactylation, remains unclear and warrants further structural investigation using techniques such as cryo‐electron microscopy (cryo‐EM) and nuclear magnetic resonance (NMR) [53, 54]. Additionally, while GCN5 appears to mediate K11 lactylation, the broader upstream network, including kinases and pathological signals regulating these PTMs, is not fully delineated. Our reliance on in vitro and murine models may also limit translational relevance, necessitating validation in human tissues or clinically relevant stroke models.

In conclusion, this study elucidates a dual‐modification mechanism by which K11 lactylation and S199 phosphorylation cooperatively regulate CKB activity and neuroprotection in cerebral I/R. The interplay between these PTMs highlights a novel layer of enzymatic control with broad implications for neuronal metabolism and injury repair. Our findings open new avenues for targeting post‐translational modification crosstalk in neuroprotective strategies and deepen our understanding of how metabolic signals are transduced into functional outcomes through precise protein modifications.

## 4 Material and Methods

### 4.1 Intravenous Injection of Adeno‐Associated Virus (AAV) in Mice

All animal procedures were performed in compliance with institutional animal care guidelines. Prior to viral (Table S1) transduction, mice were transferred to an anesthesia induction chamber, where general anesthesia was induced with 2% isoflurane during the entire surgical procedure. The mouse tail was sterilized with 75% ethanol before injection to avoid contamination. A heating pad was continuously applied throughout and after the operation to maintain a stable body temperature in mice. 29‐gauge insulin syringes (Ultra‐Fine Insulin Syringe, BD, USA) were used to inject 3 × 10^11^ vg / mouse. All subsequent modeling and analyses were conducted at 3 weeks post AAV transfection.

### 4.2 MCAO/R Model

Transient focal cerebral ischemia was induced in male C57BL/6 mice (7–8 weeks old, 22–24 g) via MCAO/R as previously described [55]. Mice were anesthetized with 2% isoflurane (RWD, China), and a midline cervical incision was made to expose the left common carotid artery (CCA), external carotid artery (ECA), and internal carotid artery (ICA). The CCA was ligated distally, and two ligatures were placed on the ECA, one at its distal end and the other at the ECA‐ICA bifurcation. A silicon‐coated filament (MSMC21B100PK50, RWD, China) was inserted into the ICA through a small incision in the ECA to occlude the middle cerebral artery. After 2 h, the filament was removed to initiate reperfusion. Rectal temperature was maintained at 37°C throughout surgery. Sham‐operated mice underwent identical procedures without filament insertion. Cerebral infarction and edema were assessed by TTC staining 24 h post‐reperfusion. Animals were randomly assigned to groups; experimenters performing behavioral tests and histology were blinded to allocation.

### 4.3 Lactate Assay

Cortical tissues or cells were homogenized in lysis buffer and sonicated (200 W, 2 s on / 5 s off for 5 min) on ice. After centrifugation at 12 000 g for 10 min at 4°C, lactate levels in the supernatant were quantified using a Lactate Colorimetric Assay Kit (ab65331, Abcam) according to the manufacturer's protocol.

### 4.4 Proteomics

Protein digestion and peptide preparation: Samples were ground in liquid nitrogen and lysed in a buffer containing 8 m urea and 1% protease inhibitor. The lysates were sonicated, reduced with dithiothreitol, alkylated with iodoacetamide, and digested with trypsin.

LC‐MS/MS analysis for proteomics: The tryptic peptides were dissolved in solvent A, directly loaded onto a home‐made reversed‐phase analytical column (25 cm length, 100 µm i.d.). The mobile phase consisted of solvent A (0.1% formic acid, 2% acetonitrile in water) and solvent B (0.1% formic acid in acetonitrile). Peptides were separated with the following gradient: 0–70 min, 6%–24% B; 70–82 min, 24%–35% B; 82–86 min, 35%‐80% B; 86–90 min, 80% B, and all at a constant flow rate of 450 nL/min on a NanoElute UHPLC system (Bruker Daltonics). The peptides were subjected to a capillary source followed by the timsTOF Pro2 mass spectrometry. The electrospray voltage applied was 1.6 kV. Precursors and fragments were analyzed at the TOF detector, with a MS/MS scan range from 100 to 1700. The timsTOF Pro2 was operated in parallel accumulation serial fragmentation (PASEF) mode. Precursors with charge states 0–5 were selected for fragmentation, and 10 PASEF‐MS/MS scans were acquired per cycle. The dynamic exclusion was set to 30 s.

Database search for proteomics: The resulting MS/MS data were processed using MaxQuant search engine (v.1.6.15.0). Tandem mass spectra were searched against the Mus_musculus_10090_SP_20230103.fasta (17132 entries) concatenated with a reverse decoy database. Trypsin/P was specified as a cleavage enzyme allowing up to 2 missing cleavages. The mass tolerance for precursor ions was set as 20 ppm in the first search, and 20 ppm in the main search, and the mass tolerance for fragment ions was set as 20 ppm. Carbamidomethyl on Cys was specified as a fixed modification, and acetylation on protein N‐terminal and oxidation on Met were specified as variable modifications. FDR was adjusted to < 1%.

### 4.5 Lactylomics

Protein digestion and peptide preparation: Samples were ground in liquid nitrogen and lysed in a buffer containing 8 m urea and 1% protease inhibitor. The lysates were sonicated, reduced with dithiothreitol, alkylated with iodoacetamide, and digested with trypsin.

Pan antibody‐based PTM enrichment: To enrich modified peptides, tryptic peptides dissolved in NETN buffer (100 mm NaCl, 1 mm EDTA, 50 mm Tris‐HCl, 0.5% NP‐40, pH 8.0) were incubated with pre‐washed antibody beads (Lot number PTM1404, PTM Bio) at 4°C overnight with gentle shaking. Then the beads were washed four times with NETN buffer and twice with H_2_O. The bound peptides were eluted from the beads with 0.1% trifluoroacetic acid. Finally, the eluted fractions were combined and vacuum‐dried. The resulting peptides were desalted with C18 ZipTips (Millipore) according to the manufacturer's instructions for LC‐MS/MS analysis.

LC‐MS/MS analysis for lactylomics: Pan antibody‐based PTM enrichment peptides were dissolved in solvent A, directly loaded onto a home‐made reversed‐phase analytical column (25 cm length, 100 µm i.d.). The mobile phase consisted of solvent A (0.1% formic acid, 2% acetonitrile in water) and solvent B (0.1% formic acid in acetonitrile). Peptides were separated with the following gradient: 0–42 min, 6%–22% B; 42–52 min, 22%–30% B; 52–56 min, 30%–80% B; 56–60 min, 80% B, and all at a constant flow rate of 450 nL/min on a NanoElute UHPLC system (Bruker Daltonics). The peptides were subjected to a capillary source followed by the timsTOF Pro2 mass spectrometry. The electrospray voltage applied was 1.50 kV. Precursors and fragments were analyzed at the TOF detector, with a MS/MS scan range from 100 to 1700. The timsTOF Pro2 was operated in parallel accumulation serial fragmentation (PASEF) mode. Precursors with charge states 0–5 were selected for fragmentation, and 10 PASEF‐MS/MS scans were acquired per cycle. The dynamic exclusion was set to 24 s.

Database search for lactylomics: The resulting MS/MS data were processed using MaxQuant search engine (v.1.6.15.0). Tandem mass spectra were searched against the Mus_musculus_10090_SP_20230103.fasta (17132 entries) concatenated with a reverse decoy database. Trypsin/P was specified as a cleavage enzyme allowing up to 4 missing cleavages. The mass tolerance for precursor ions was set as 20 ppm in the first search, and 20 ppm in the main search, and the mass tolerance for fragment ions was set as 20 ppm. Carbamidomethyl on Cys was specified as a fixed modification, and acetylation on protein N‐terminal and oxidation on Met and lactylation on Lys were specified as variable modifications. FDR was adjusted to < 1%.

### 4.6 Phosphoproteomic

Protein digestion and peptide preparation: Samples were ground in liquid nitrogen and lysed in a buffer containing 8 m urea and 1% protease inhibitor. The lysates were sonicated, reduced with dithiothreitol, alkylated with iodoacetamide, and digested with trypsin.

Bio‐material‐based PTM enrichment (for phosphorylation): Peptide mixtures were first incubated with IMAC microspheres suspension with vibration in loading buffer (50% acetonitrile/0.5% acetic acid). To remove the non‐specifically adsorbed peptides, the IMAC microspheres were washed with 50% acetonitrile/0.5% acetic acid and 30% acetonitrile/0.1% trifluoroacetic acid, sequentially. To elute the enriched phosphopeptides, the elution buffer containing 10% NH_4_OH was added, and the enriched phosphopeptides were eluted with vibration. The supernatant containing phosphopeptides was collected and lyophilized for LC‐MS/MS analysis.

LC‐MS/MS analysis for phosphoproteomic: Bio‐material‐based PTM enrichment peptides were dissolved in solvent A, directly loaded onto a home‐made reversed‐phase analytical column (25‐cm length, 100 µm i.d.). The mobile phase consisted of solvent A (0.1% formic acid, 2% acetonitrile in water) and solvent B (0.1% formic acid in acetonitrile). Peptides were separated with the following gradient: 0–76 min, 2%–22% B; 76–82 min, 22%–35%B; 82–86 min, 35%–90%B; 86–90 min, 90%B, and all at a constant flow rate of 450 nL/min on a NanoElute UHPLC system (Bruker Daltonics). The peptides were subjected to a capillary source followed by the timsTOF Pro2 mass spectrometry. The electrospray voltage applied was 1.7 kV. Precursors and fragments were analyzed at the TOF detector, with a MS/MS scan range from 100 to 1700. The timsTOF Pro2 was operated in parallel accumulation serial fragmentation (PASEF) mode. Precursors with charge states 0–5 were selected for fragmentation, and 10 PASEF‐MS/MS scans were acquired per cycle. The dynamic exclusion was set to 24 s.

Database search for phosphoproteomic: The resulting MS/MS data were processed using MaxQuant search engine (v.1.6.15.0). Tandem mass spectra were searched against the Mus_musculus_10090_SP_20230103.fasta (17132 entries) concatenated with a reverse decoy database. Trypsin/P was specified as a cleavage enzyme allowing up to 2 missing cleavages. The mass tolerance for precursor ions was set as 20 ppm in the first search, and 20 ppm in the main search, and the mass tolerance for fragment ions was set as 20 ppm. Carbamidomethyl on Cys was specified as a fixed modification, and acetylation on protein N‐terminal, oxidation on Met, and phosphorylation on Ser, Thr, Tyr were specified as variable modifications. FDR was adjusted to < 1%.

### 4.7 TTC Staining

Harvested brains were coronally sectioned and incubated in 1% TTC solution (T8170, Solarbio, China) for 30 min at 37°C. After washing with PBS, slices were fixed in 4% paraformaldehyde (PFA) for 6 h, and infarct areas were quantified using imageJ analysis.

### 4.8 Motor Behavior Tests

Balance beam, ladder rung, and rotarod tests were performed at baseline and at multiple time points post‐MCAO/R for up to 4 weeks to evaluate motor function recovery.

For the balance beam, mice were trained on the balance beam for 3 days prior to MCAO/R surgery. During behavioral testing, the number of right hindlimb slips was recorded over three consecutive trials. The scores were full score (10) minus the number of foot slips. A score of 0 was assigned if the mouse failed to cross or fell off the beam.

For the ladder rung, mice were assessed for missteps while traversing the ladder rung. The score was calculated as 10 minus the number of wrong steps, with a minimum score of 0 if the mouse could not complete the task.

For the rotarod test, mice were pre‐trained on the accelerating rotarod (30 rpm) before MCAO/R. During testing, latency to fall was recorded over three trials (30 rpm, maximum cutoff: 300 s). The final score represented the mean time spent on the rotarod across trials.

### 4.9 Nissl staining

Brain sections were stained using Nissl Stain Solution (G1434, Solarbio, China). Tissues were incubated with methylene blue at 65°C for 10 min, differentiated with Nissl differentiation solution for 3 min, and treated with ammonium molybdate for 5 min. After rinsing, sections were imaged using an inverted microscope (Leica, Germany).

### 4.10 Immunofluorescence

Mice were perfused with PBS followed by 10% formalin. Brains were post‐fixed overnight at 4°C and cryoprotected in 30% sucrose for 48 h. Tissues embedded in OCT (SAKURA, Japan) were sectioned (15 µm) using a cryostat (Leica, Germany). Sections were permeabilized (0.25% Triton X‐100), blocked in 2% BSA, and incubated overnight at 4°C with primary antibodies: anti‐MAP2 (Abcam, ab5392, 1:500), anti‐GFAP (CST, 3670, 1:500), anti‐Iba1 (ABclonal, A27316, 1:200), anti‐Active Caspase‐3 (BD, 559665, 1:250), anti‐NeuN (Proteintech, 66836‐1‐Ig, 1:300), or anti‐Pan‐Kla (PTMab, PTM‐1401RM, 1:75). Goat anti‐Rabbit IgG (H+L) (A‐11008, Invitrogen, USA) and Goat anti‐Mouse IgG (H+L) (A‐21235, Invitrogen, USA) were applied for 1 h at room temperature. Nuclei were counterstained with DAPI. Images were acquired using a spinning disk confocal microscope (Andor Technology, UK).

### 4.11 Cultivation of Primary Neuron Cells

Primary neural cells were prepared from the cortex of mouse embryos under sterile conditions (obtained from pregnant C57BL/6 mice). The harvested embryonic tissues were immediately transferred to pre‐cooled calcium‐ and magnesium‐free Hanks’ balanced salt solution (CMF‐HBSS). Under a dissecting microscope, the cortical tissues were exposed, and the overlying meningeal membranes and superficial blood vessels were thoroughly and carefully removed. The isolated cortical tissues were digested with 0.05% trypsin‐EDTA solution at 37°C for 15 min, with gentle shaking every 5 min during the digestion period. After enzymatic digestion, the tissues were washed three times with CMF‐HBSS and softly triturated approximately ten times in CMF‐HBSS to prepare single‐cell suspensions, with strict avoidance of air bubble generation during trituration. The resulting cell supernatant was filtered, and the cell suspension was centrifuged at 1000 rpm for 10 min at 4°C. After discarding the supernatant, the cell pellet was resuspended in DMEM medium. Cell counting and viability detection were performed via trypan blue staining. Subsequently, cells were seeded onto PLL (poly‐L‐lysine) pre‐coated culture plates at gradient densities: 5 × 10^4^ cells/well for 12‐well plates and 2 × 10^5^ cells/well for 6‐well plates. All cells were incubated at 37°C in a humidified atmosphere containing 5% CO_2_. A complete medium exchange with Neurobasal medium was performed 3 h after cell seeding. For routine maintenance, half‐volume medium replacement was conducted twice a week. Following 7 days of adherent culture, the primary cortical neurons were subjected to AAV infection and oxygen‐glucose deprivation/reoxygenation (OGD/R) modeling.

### 4.12 AAV Transduction of Primary Neuron Cells

Cultured primary mouse cortical and hippocampal neurons were subjected to AAV transduction. Purified AAV vectors (Table S2) were applied to neuronal cultures at optimized dosages according to plate specifications: 5 × 10 ^9^ vg / well for 12‐well plates and 2 × 10 ^10^ vg / well for 6‐well plates. The culture medium was partially replaced with pre‐warmed fresh medium before viral treatment. AAV vectors were gently added to each well with mild shaking to ensure even distribution. Neurons were incubated with AAV at 37°C under 5% CO_2_. The culture medium was retained for the first 24 h post‐transduction and then completely refreshed to eliminate unbound viral particles. Neuron cells were harvested for subsequent experiments at 72 h after AAV infection.

### 4.13 OGD/R model

Neuro‐2a cells (Procell, Wuhan, China) were maintained in MEM medium supplemented with 10% FBS and 1% penicillin‐streptomycin at 37°C and 5% CO_2_. OGD/R was induced by replacing media with glucose‐free, serum‐free MEM and incubating in a hypoxia chamber (0.3% O_2_, 5% CO_2_, 94.7% N_2_) for 4 h, followed by reoxygenation under normoxic conditions for 24 h. Control cells were maintained under normoxia for the same duration.

### 4.14 Plasmid Construction and Transfection

Full‐length coding sequences of CKB, P300, CBP, GCN5, PCAF, Sirt1/2/3/5 were cloned into pCDNA3.1 vectors containing 3×Flag or 3×HA tags via homologous recombination. Constructs were verified by sequencing. Transfections were performed using Lipofectamine 3000 (L3000015, Invitrogen, USA), and cells were harvested 24–48 h post‐transfection.

### 4.15 Cell Viability

Viability was assessed using CCK‐8 (C0042, Beyotime, China). Following OGD/R, 2 × 10^3^ cells/well were seeded in 96‐well plates, treated with 10 µL CCK‐8 solution for 1 h at 37°C, and absorbance measured at 450 nm.

### 4.16 Creatine Kinase Activity

CK activity was measured using a Creatine Kinase Activity Assay Kit (BC1145, Solarbio, China) according to the manufacturer's protocol. The assay is based on coupled enzymatic reactions resulting in NADPH generation, monitored by absorbance at 340 nm.

### 4.17 Creatine Assay

Creatine content was determined using a commercial kit (BC4925, Solarbio, China). Absorbance was measured at 530 nm using a plate reader (Spectra Max M5, Molecular Devices, USA).

### 4.18 ATP Assay

After 24 h of reperfusion, cells were harvested, and ATP level was measured with ATP Assay Kit (S0026, Beyotime, China) according to the manufacturer's protocol.

### 4.19 ROS Detection

Intracellular ROS was evaluated using DCFH‐DA (D6470, Solarbio, China). After incubation for 15 min at 37°C, cells were fixed with 4% PFA and imaged under a spinning disk confocal microscope. ROS signals were quantified using ImageJ from 10 randomly selected ROIs per condition.

### 4.20 Analysis of Mitochondrial Membrane Potential

Mitochondrial function was assessed by TMRE (C2001S, Beyotime, China) in accordance with the manufacturer's instructions. After 24 h of reperfusion, the Neuro‐2a cells were washed three times with 37°C MEM medium and incubated in 500 µL of 1 ×TMRE solution at 37°C for 20 min in the dark. Nuclei were stained with Hoechst‐33342. The cells were washed six times with 37°C MEM medium and imaged under a spinning disk confocal microscope (Andor Technology, UK). Images were analyzed using ImageJ software.

### 4.21 Seahorse Assay

Seahorse assay was adopted to detect mitochondrial respiratory capacity. The Seahorse XF Cell Mito Stress Test Kit (103015‐100, Agilent, Delaware, USA) was used to directly measure the oxygen consumption rate (OCR) using an Agilent Seahorse Xfe24 Extracellular Flux Analyzer (Agilent, USA). Cells were seeded at a density of 2 × 104 cells per wellin Seahorse XF cell culture 24‐well microporous plates (102342‐100, Agilent, Delaware, USA) and subjected to the indicated treatments. The sensor cartridge was hydrated overnight at 37°C in a CO_2_‐free incubator using Seahorse XF calibration solution. During the assay, oligomycin A, FCCP, and antimycin A were sequentially injected to final concentrations of 1.5, 0.5, and 2 µm, respectively. OCR was recorded and analyzed according to the manufacturer's instructions.

### 4.22 MDA assay

MDA levels were assessed using an MDA Assay Kit (BC0025, Solarbio, China). Following OGD/R and lysis, samples were heated with MDA reagent at 100°C and absorbance read at 532 nm. MDA content was calculated based on standard curves.

### 4.23 Immunoprecipitation

For HA/Falg‐Nanoab IP, cells were lysed with pre‐chilled PBS and lysed in ice‐cold IP lysis buffer (50 mm Tris‐HCl, 150 mm NaCl, 1% Triton X‐100, 2 mm EDTA, 1 mm PMSF, and 1× complete protease inhibitor cocktail) and incubated with HA‐Nanoab‐Magnetic Beads (HNM‐25‐1000, Lablead, China) or DYKDDDDK‐Nanoab‐Magnetic beads (FM0500, Lablead, China) overnight at 4°C with gentle rotation.

For endogenous CKB‐IP, fresh cell and tissue samples were rinsed with pre‐chilled PBS and lysed in ice‐cold IP lysis buffer for 30 min at 4°C, followed by centrifugation at 12 000 rpm for 15 min at 4°C to collect soluble total protein supernatants. To eliminate non‐specific binding, validated rabbit anti‐CKB antibody (Abcam, ab92452, USA) was pre‐adsorbed with Protein A/G Magnetic beads in advance to form stable antibody‐bead complexes. After protein quantification and equal normalization of all clarified lysates, the prepared CKB antibody‐Protein A/G bead complexes were incubated with normalized protein samples overnight at 4°C with gentle rotation.

The bead‐protein immune complexes were then harvested, thoroughly washed five times with pre‐chilled IP lysis buffer at 4°C to remove unbound and non‐specific contaminants, and finally boiled in 1× SDS loading buffer at 95°C for 10 min to fully elute and denature enriched CKB proteins for subsequent Western blot analysis.

### 4.24 Western Blotting

Total proteins were extracted using RIPA buffer with protease, phosphatase, and deacetylase inhibitors. Samples were separated by SDS‐PAGE (7.5%–12.5%) and transferred to PVDF membranes. Blots were probed with anti‐β‐actin (Proteintech, 66009‐1‐Ig, 1:5000), anti‐DYDDDDK (Proteintech, 20543‐1‐AP, 1:5000), anti‐CKB (Proteintech, 66764‐1‐Ig, 1:2000), anti‐GCN5 (Proteintech, 66575‐1‐Ig, 1:1000), anti‐HA (ABclonal, AE105, 1:5000), anti‐Pan Kla (PTMab, PTM‐1401RM, 1:1000), anti‐p‐CKB S199 (Invitrogen, PA5‐106621, 1:1000), anti‐Active‐Caspase 3 (BD, 559665, 1:1000) antibodies overnight at 4°C with gentle rotation. After incubation with anti‐rabbit nanobody‐HRP (RN0100, Lablead, China) or anti‐mouse nanobody‐HRP (MN0100, Lablead, China) at room temperature for 2 h, the signal intensities were visualized by a ECL chemiluminescence kit (WBKLS0500, Merck, Germany).

### 4.25 q‐PCR

Total RNA was extracted with TRIzol and reverse transcribed using a HiScript III 1st Strand cDNA Synthesis Kit (R312‐01, Vazyme, China). qPCR was conducted on a CFX96 system (Bio‐Rad, USA) using SYBR Green (Q321‐02, Vazyme, China) and specific primers (Table S2). Cycling conditions: 95°C for 30 s; 40 cycles of 95°C for 10 s, 60°C for 30 s. Gene expression was normalized to β‐actin using the 2^−^^ΔΔCt^ method.

### 4.26 Molecular Dock

Molecular docking was performed to evaluate the binding between mouse CKB (UniProt: Q04447) and GCN5 (UniProt: Q9JHD2), and to determine how S164 and S199 phosphorylation of CKB affects their interaction. All structural processing and docking simulations were carried out using AutoDock Vina 1.2.6, and structural visualization was performed with PyMOL 2.5. After removing water molecules and redundant heteroatoms, polar hydrogen atoms and Gasteiger charges were assigned to optimize protein structures. Site‐specific phosphorylation modifications were artificially introduced at the S164 and S199 sites of CKB. CKB, CKB S164, and CKB S199 were separately defined as ligands, while GCN5 was set as the receptor. The grid box was centered to cover the potential interaction interface of GCN5, and rigid‐body docking was conducted with standard parameters. The optimal conformation of each group was selected according to the lowest binding free energy. Differences in hydrogen bonding and hydrophobic interactions between GCN5 and wild‐type or phosphorylated CKB were finally analyzed to assess phosphorylation‐dependent binding alterations.

### 4.27 Statistical Analysis

Data are expressed as mean ± SD from three independent experiments. Statistical analyses were conducted using GraphPad Prism 9. Two‐group comparisons employed an unpaired Student's *t*‐test; multiple group comparisons used one‐way ANOVA with Tukey's post hoc test. ^*^ *p* < 0.05, ^**^ *p* < 0.01, ^***^ *p* < 0.001 were considered statistically significant.

## Funding

This study was supported by the National Natural Science Foundation of China (32571174), the Key Project of Research and Development of Hubei Province (2022BCE049), Fundamental Research Funds for the Central Universities (2662022JC002), and the Youth Fund of National Natural Science Foundation of China (32400815).

## Ethics

The animal study protocol were approved by the Institutional Animal Care and Use Committee of Huazhong Agricultural University (reference NO. HZAUMU‐2023‐0063).

## Conflicts of Interest

The authors declare no conflicts of interest.

## Supporting information




**Supporting File**: advs76641‐sup‐0001‐SuppMat.docx.

## Data Availability

The mass spectrometry‐based proteomics, lactylomics, and phosphoproteomics datasets have been deposited in the ProteomeXchange Consortium via the PRIDE partner repository under the accession numbers PXD067754, PXD067755, and PXD067636, respectively.
