# Supplementary material for: Phosphorylation‐Facilitated CKB Lactylation At K11 By GCN5 Enhances Creatine Kinase Activity and Mitigates Neuronal Damage After Cerebral Ischemia‐Reperfusion
Source: Adv Sci (Weinh). 2026 Jul 17:e16663. Online ahead of print. doi: 10.1002/advs.202516663 (PMC13379269; doi:10.1002/advs.202516663)
Supplement: Supplementary file 1 — Supporting File: advs76641‐sup‐0001‐SuppMat.docx. [file ADVS-9999-e16663-s001.docx]

**
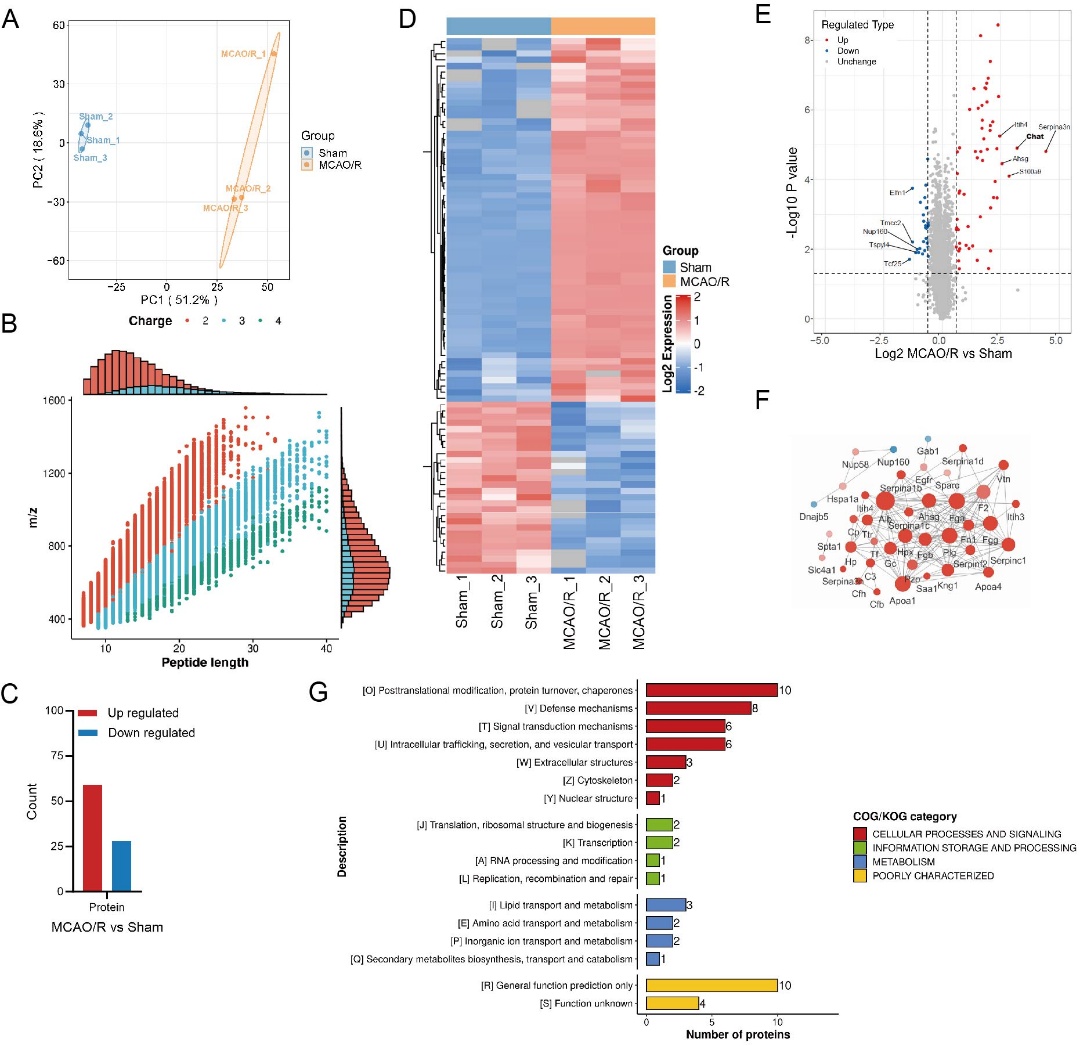
Supplementary Figures**

**Supplementary figure 1. Global proteome changes in ischemic cortex after MCAO/R.**

(A) PCA of protein intensities shows clear separation between sham and MCAO/R samples.

(B) Quality control scatter of peptide length versus m/z with charge-state distribution.

(C) Numbers of up- and downregulated proteins in MCAO/R versus sham using the predefined cutoffs.

(D) Heat map of differentially expressed proteins (row-scaled log_2_ intensities) across biological replicates.

(E) Volcano plot highlighting significantly altered proteins in MCAO/R relative to sham.

(F) Protein-protein interaction (PPI) network of dysregulated proteins illustrating clustered modules.

(G) COG/KOG functional classification of altered proteins, with bars grouped by high-level categories.

**
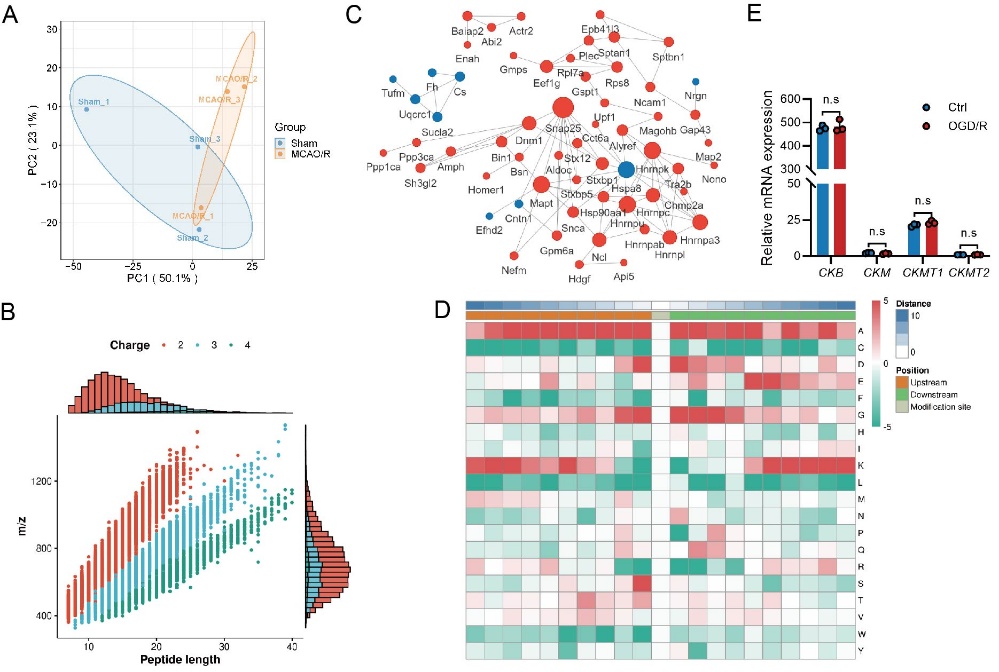
Supplementary figure 2. Lactylome profiling in ischemic cortex and cells.**

(A) PCA of lactylated peptide intensities distinguishes sham from MCAO/R cortices.

(B) QC plot of lactylated peptide length versus m/z with charge distribution.

(C) PPI network of proteins bearing lysine lactylation sites detected in cortex.

(D) Sequence context heat map showing residue enrichment flanking lactyl-lysine sites (positions upstream/downstream relative to the modification).

(E) q-PCR analysis of creatine kinase isoforms (*CKB, CKM, CKMT1, CKMT2*) in Neuro-2a cells under control and OGD/R conditions shows no significant transcriptional changes (n.s.), indicating that subsequent changes in CK activity arise primarily from post-translational regulation. n = 3 per group.

Data are presented as mean ± SEM. Statistical significance was determined by one-way ANOVA followed by Tukey's post hoc test. **p* < 0.05, ***p* < 0.01, ****p* < 0.001.

**
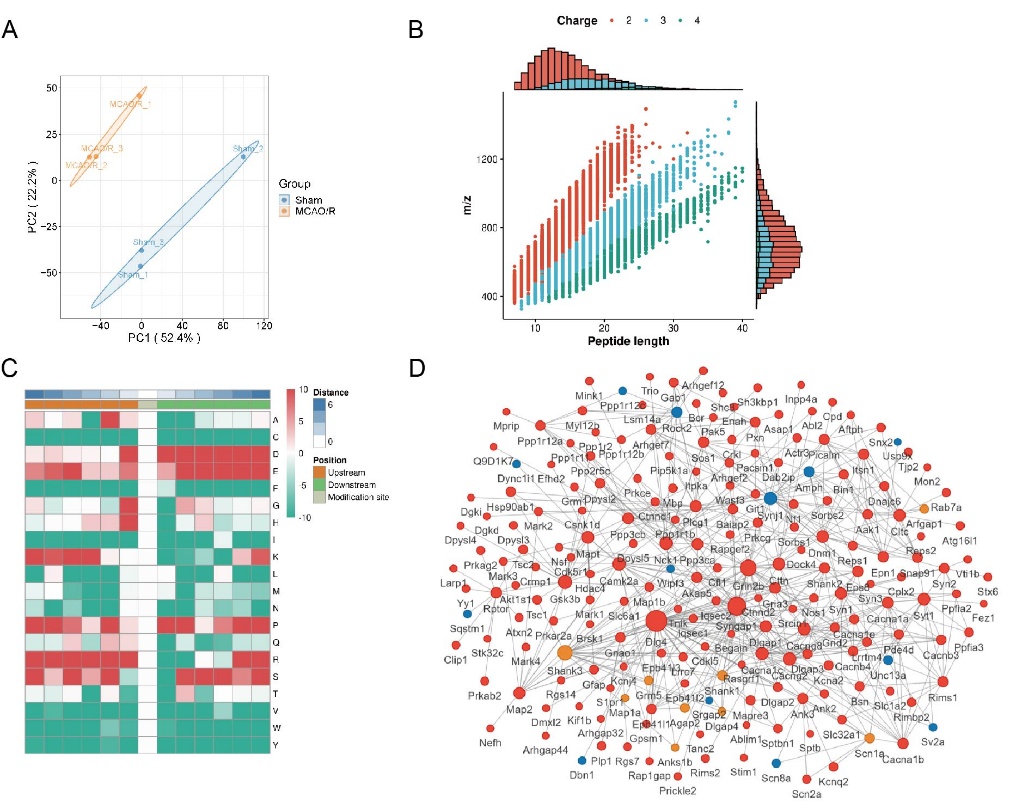
Supplementary figure 3.** **Phosphoproteome features in ischemic cortex**.

(A) PCA of phosphopeptide intensities separates sham and MCAO/R samples.

(B) QC plot of phosphopeptide length versus m/z with charge distribution.

(C) Sequence context heat map depicting residue enrichment around phosphorylation sites.

(D) PPI network of phosphoproteins altered by MCAO/R highlighting hub nodes related to neuronal signaling and stress responses.

**
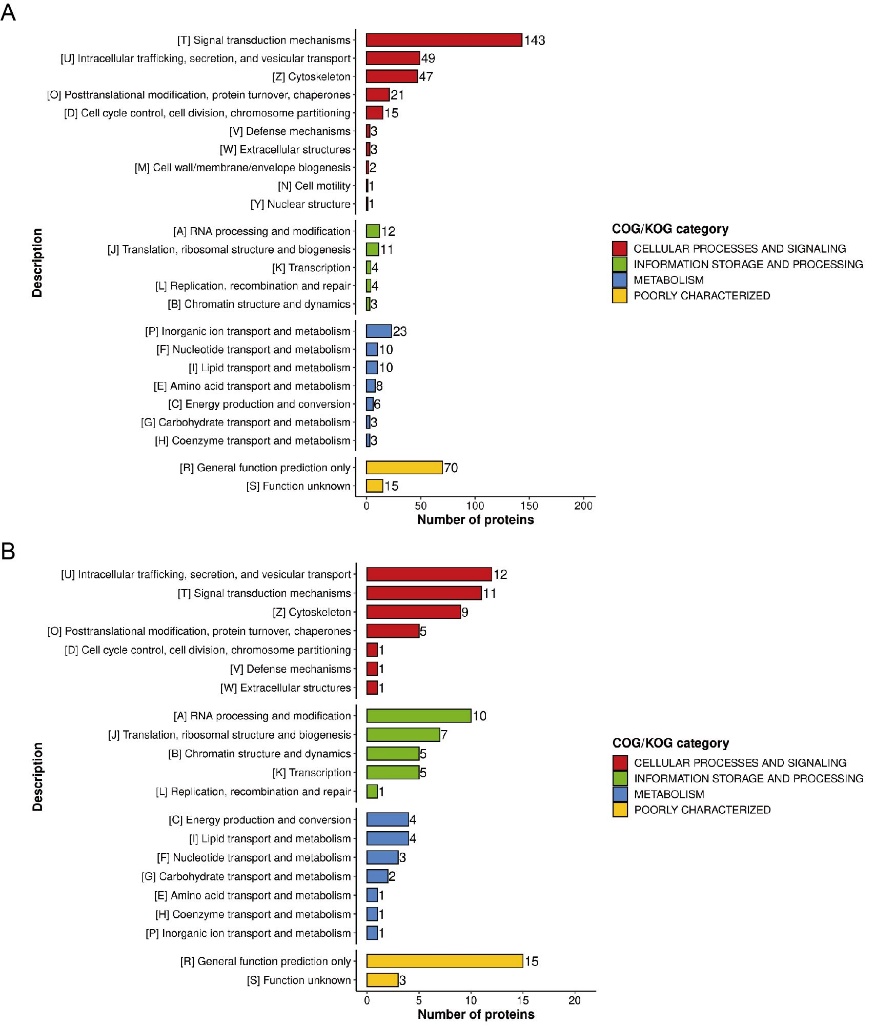
Supplementary figure 4.** **Functional categorization links modification programs to shared biology**.

(A) COG/KOG functional classification of lactylated proteins detected in cortex, showing prominent representation in signal transduction and intracellular trafficking.

(B) COG/KOG functional classification of phosphorylated proteins detected in cortex, indicating convergence on vesicular transport, signaling, and information-processing categories.


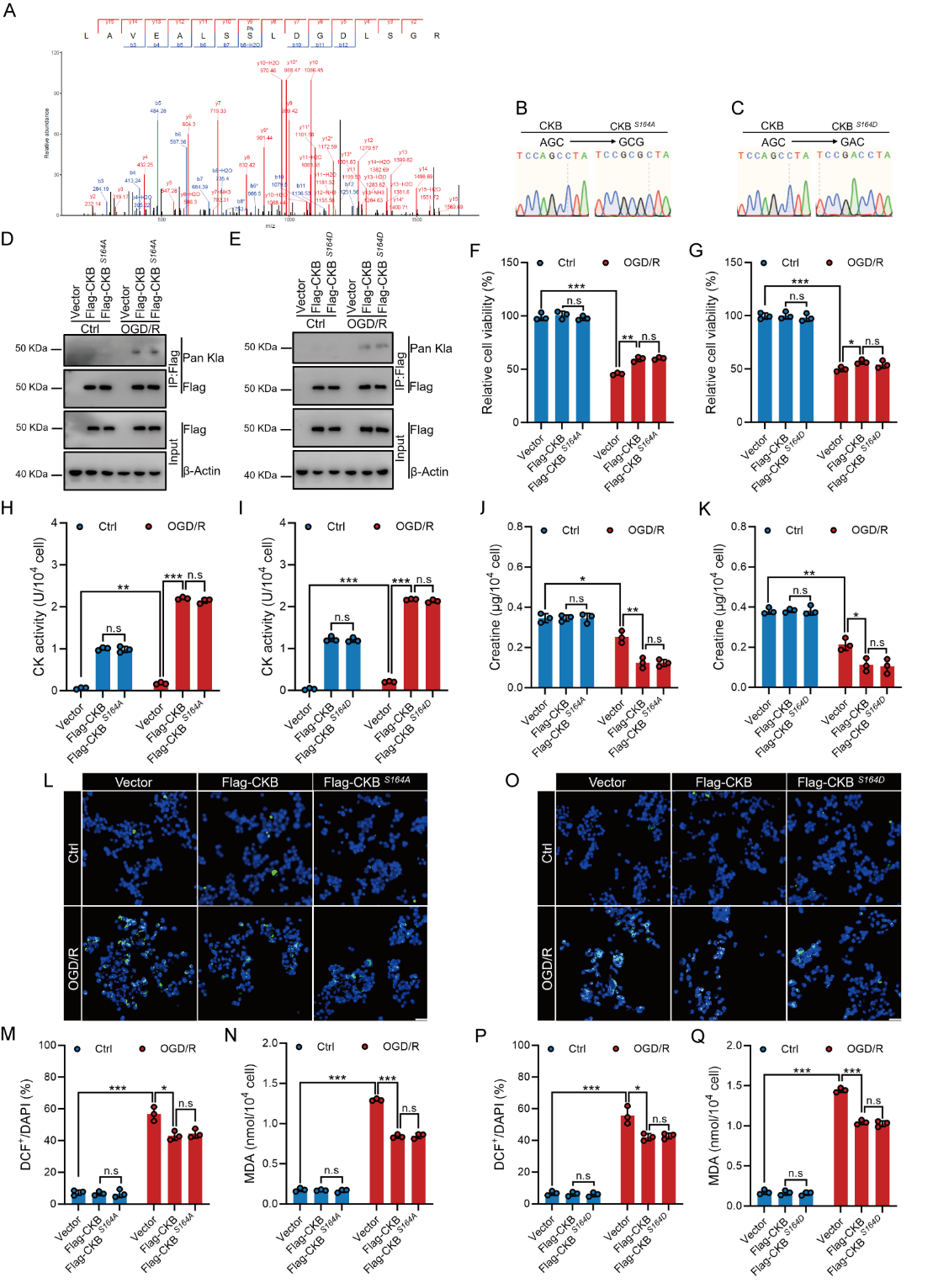
**Supplementary** **figure 5. CKB S164 phosphorylation did not affect CK activity and OGD/R-induced oxidative stress.**

(A) Mass spectrometry spectrum identifying phosphorylated CKB peptides in cortical tissues of MCAO/R mice.

(B-C) Sanger sequencing chromatograms validating CKB and CKB *^S164A^*, CKB *^S164D^* constructs.

(D-E) IP combined with western blot analysis of Pan-Kla in Neuro-2a cells expressing Flag-CKB, Flag-CKB *^S164A^* or Flag-CKB *^S164D^* in Neuro-2a cells.

(F-G) Quantitative assessment of cell viability following normoxia or OGD/R treatment in Neuro-2a cells expressing Flag-CKB, Flag-CKB *^S164A^* or Flag-CKB *^S164D^*. n = 3 per group.

(H-I) Quantitative assessment of CK enzymatic activity following normoxia or OGD/R treatment in Neuro-2a cells expressing Flag-CKB, Flag-CKB *^S164A^* or Flag-CKB *^S164D^*. n = 3 per group.

(J-K) Quantitative assessment of cellular creatine following normoxia or OGD/R treatment in Neuro-2a cells expressing Flag-CKB, Flag-CKB *^S164A^* or Flag-CKB *^S164D^*. n = 3 per group.

(L-M) Representative images (L) and quantification (M) of ROS (DCFH-DA staining) following normoxia or OGD/R treatment in Neuro-2a cells expressing Flag-CKB and Flag-CKB *^S164A^*. n = 3 per group. Scale bar: 50 μm.

(N) The quantification of MDA level following OGD/R in Neuro-2a cells expressing Flag-CKB, Flag-CKB *^S164A^* n = 3 per group.

(O-P) Representative images (O) and quantification (P) of ROS (DCFH-DA staining) following normoxia or OGD/R treatment in Neuro-2a cells expressing Flag-CKB and Flag-CKB *^S164D^*. n = 3 per group. Scale bar: 50 μm

(Q) The quantification of MDA level following OGD/R in Neuro-2a cells expressing Flag-CKB, Flag-CKB *^S164D^*. n = 3 per group.

Data are presented as mean ± SEM. Statistical significance was determined by one-way ANOVA followed by Tukey's post hoc test. **p* < 0.05, ***p* < 0.01, ****p* < 0.001.

**
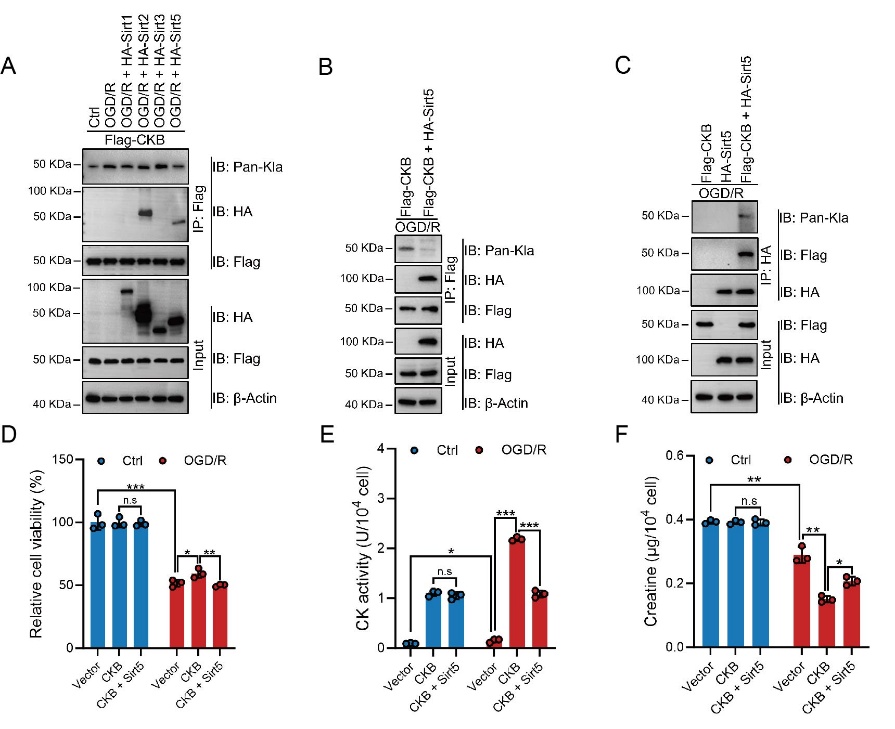
Supplementary figure 6. Sirtuin screen identifies Sirt5 as the predominant delactylase counteracting CKB-driven protection.**

(A) Different delactyltransferases were screened by IP assays. Flag-CKB was co-expressed with HA-tagged Sirt1, Sirt2, Sirt3, or Sirt5 in Neuro-2a cells; lysates were subjected to IP and immunoblot for Pan-Kla, HA and Flag.

(B-C) IP assays showing Sirt5-dependent delactylation of CKB. Flag-CKB or Flag-CKB + HA-Sirt5 were expressed in Neuro-2a cells (B); Flag-CKB, HA- Sirt5 or Flag-CKB + HA-Sirt5 were expressed in Neuro-2a cells (C); lysates were subjected to IP and immunoblot for Pan-Kla, HA and Flag.

(D-F) Neuro-2a cells were transfected with Flag-CKB or Flag-CKB + HA-Sirt5 and subjected to normoxia or OGD/R; Cell viability (D), CK activity (E) and creatine levels (F) were examined and quantified. n = 3 per group.

Data are presented as mean ± SEM. Statistical significance was determined by one-way ANOVA followed by Tukey's post hoc test. **p* < 0.05, ***p* < 0.01, ****p* < 0.001.


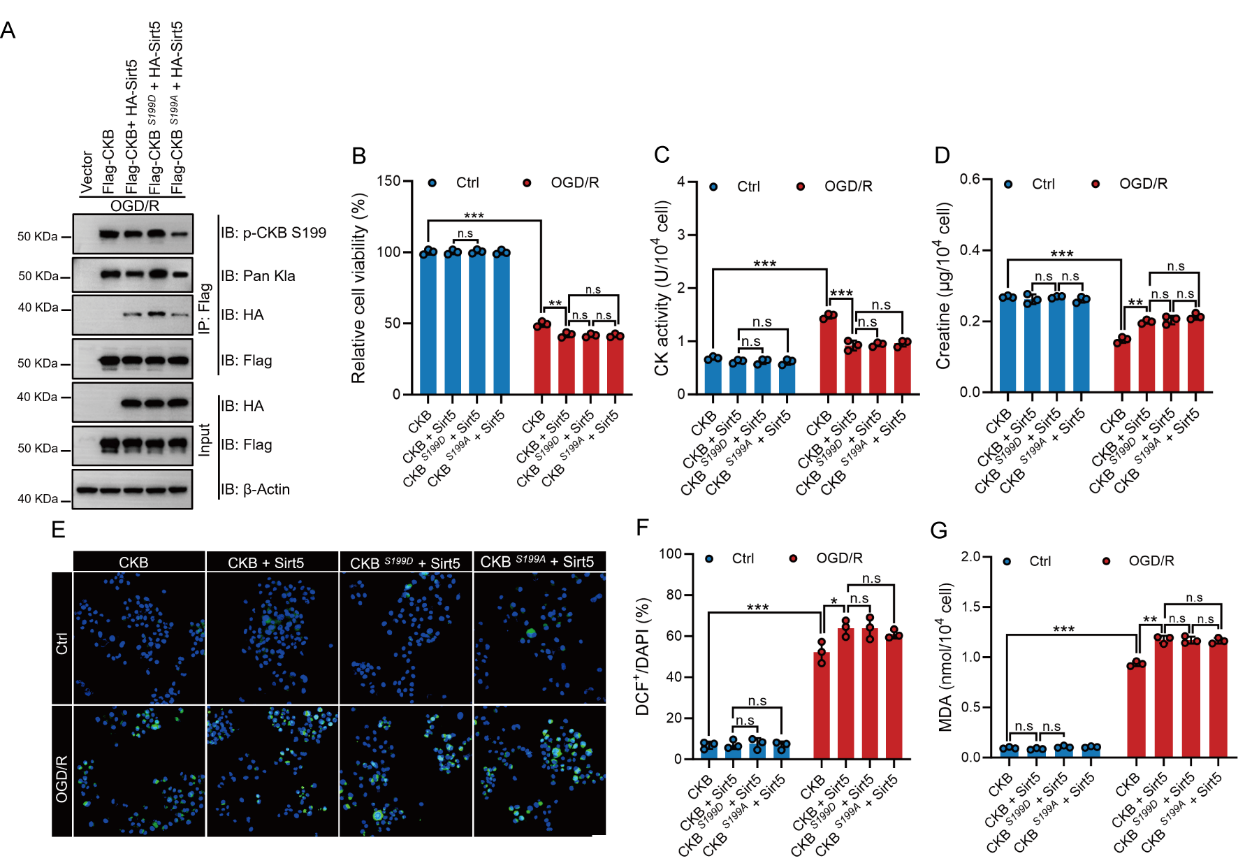
**Supplementary figure 7. CKB S199 phosphorylation state shapes OGD/R stress phenotypes without preventing Sirt5-mediated delactylation.**

(A) Immunoblots of Flag-CKB, Flag-CKB *^S199A^* or Flag-CKB *^S199D^* co-expressed with HA-Sirt5 following normoxia or OGD/R treatment.

(B-D) Quantification of cell viability (B), CK enzymatic activity (C), and cellular creatine (D) in Flag-CKB, Flag-CKB *^S199A^* or Flag-CKB *^S199D^* co-expressed with HA-Sirt5 Neuro-2a cells following normoxia or OGD/R treatment. n = 3 per group.

(E-F) Representative images (E) and quantification (F) of intracellular ROS (DCFH-DA staining) in Flag-CKB, Flag-CKB *^S199A^* or Flag-CKB *^S199D^* co-expressed with HA-Sirt5 Neuro-2a cells following normoxia or OGD/R treatment. n = 3 per group. scale bar: 50 μm.

(G) MDA quantification of Flag-CKB, Flag-CKB *^S199A^* or Flag-CKB *^S199D^* co-expressed with HA-Sirt5 following normoxia or OGD/R treatment. n = 3 per group.

Data are presented as mean ± SEM. Statistical significance was determined by one-way ANOVA followed by Tukey's post hoc test. **p* < 0.05, ***p* < 0.01, ****p* < 0.001.


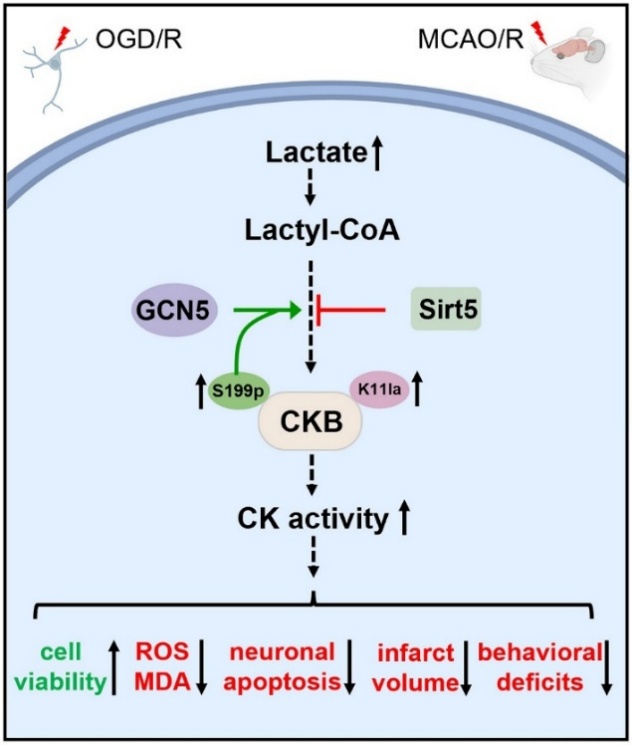
**Supplementary figure 8.** Schematic of the core mechanism: Phosphorylation-facilitated CKB K11 lactylation enhances CK activity and neuroprotection after cerebral I/R. I/R (OGD/R in vitro; MCAO/R in vivo) elevates lactate, generating lactyl-CoA (dashed arrows). In neurons, phosphorylation at CKB serine-199 (S199p) facilitates GCN5-mediated lysine-11 lactylation (K11la) on CKB (green solid arrow), whereas Sirt5 counteracts this modification (red T-bar). The resulting increase in CK activity is associated with improved cell viability and reductions in ROS/MDA, neuronal apoptosis, infarct volume, and behavioral deficits. Symbols and colors: green solid arrow, activation/promotion; red T-bar, inhibition; black dashed arrow, metabolic flow or substrate supply pathway; black up/down arrow, increase/decrease. Abbreviations: S199p, serine-199 phosphorylation; K11la, lysine-11 lactylation.

**Supplementary table 1**

AAV information

| Virus strains | Serotype | Source |
| --- | --- | --- |
| pAAV-*hSyn*-EGFP-P2A-3xFlag-WPRE | AAV2/PHP.eB | OBiO |
| pAAV-*hSyn*-EGFP-P2A-CKB-3xFlag-WPRE | AAV2/PHP.eB | OBiO |
| pAAV-*hSyn*-EGFP-P2A-CKB (*K11R*)-3xFlag-WPRE | AAV2/PHP.eB | OBiO |
| pAAV-*hSyn*-EGFP-P2A- CKB (*S199A*)-3xFlag-WPRE | AAV2/PHP.eB | OBiO |

**Supplementary table 2**

q-PCR primer sequence

| Name | Sequence (5’-3’) |
| --- | --- |
| *mβ-actin*_ Forward | GTGACGTTGACATCCGTAAAGA |
| *mβ-actin*_ Reverse | GCCGGACTCATCGTACTCC |
| *mCKB*_ Forward | AGTTCCCTGATCTGAGCAGC |
| *mCKB*_ Reverse | GAATGGCGTCGTCCAAAGTAA |
| *mCKM*_ Forward | CTGACCCCTGACCTCTACAAT |
| *mCKM*_ Reverse | CATGGCGGTCCTGGATGAT |
| *mCKMT1*_ Forward | ACCTGACCCCAGCAGTCTATG |
| *mCKMT1*_ Reverse | TTGGCTCACTTTACTGGCATC |
| *mCKMT2*_ Forward | ACACCCAGTGGCTATACCCTG |
| *mCKMT2*_ Forward | CCGTAGGATGCTTCATCACCC |
